# Integrative single‐cell analysis uncovers distinct tumour microenvironment ecotypes and immune evasion across skin cancers

**DOI:** 10.1002/ctm2.70611

**Published:** 2026-02-04

**Authors:** Lingjuan Huang, Huihui Hou, Xiyuan Zhang, Liang Dong, Wensheng Shi, Mason Liu, Jie Sun, Anil Prakash, Haoqiu Song, Shiyao Pei, Xin Li, Xiang Chen, Shenglin Mei, Mingzhu Yin

**Affiliations:** ^1^ Department of Dermatology Hunan Engineering Research Center of Skin Health and Disease Hunan Key Laboratory of Skin Cancer and Psoriasis Xiangya Hospital Central South University Changsha China; ^2^ Fralin Biomedical Research Institute Virginia Tech FBRI Cancer Research Center Washington District of Columbia USA; ^3^ Department of Radiation Oncology Nanfang hospital Southern Medical University Guangzhou China; ^4^ Clinical Research Center Medical Pathology Center Cancer Early Detection and Treatment Center and Translational Medicine Research Center Chongqing University Three Gorges Hospital Chongqing University Chongqing China; ^5^ Chongqing Technical Innovation Center for Quality Evaluation and Identification of Authentic Medicinal Herbs Chongqing China; ^6^ Chongqing University Three Gorges Hospital & Academy for Advanced interdisciplinary Technology, CQU—Ferenc Krausz Nobel Laureate Scientific Workstation Chongqing China; ^7^ School of Medicine Chongqing University Chongqing China; ^8^ Department of Urology The Second Xiangya Hospital Central South University Changsha China; ^9^ Thomas Wootton High School Rockville Maryland USA; ^10^ School of Biomedical Engineering Hainan University Sanya China; ^11^ Department of Computer Science Virginia Polytechnic Institute and State University Blacksburg Virginia USA; ^12^ Department of Dermatology The Third Xiangya Hospital Central South University Changsha China; ^13^ Department of Biomedical Sciences and Pathobiology College of Veterinary Medicine, Virginia Tech Blacksburg Virginia USA

**Keywords:** immunotherapy, melanoma, skin cancer, tumour microenvironment, tumour progression

## Abstract

**Background:**

Skin cancers, including basal cell carcinoma (BCC), squamous cell carcinoma (SCC), cutaneous melanoma (CM) and acral melanoma (AM), exhibit profound heterogeneity in clinical behaviour and therapeutic response. However, how tumour‐immune ecosystems are remodelled across skin cancer types and disease stages, and how these changes influence immune escape and treatment resistance, remain poorly understood.

**Methods:**

Here, we integrate single‐cell transcriptomics data from 102 skin cancer samples (including adjacent normal skin, early‐stage and advanced‐stage tumours), with bulk RNA‐seq prognosis cohorts, immunofluorescence staining and in vitro assays to define clinically relevant immune remodelling patterns.

**Results:**

Our analyses identify a malignant *NARS2^+^NDUFC2^+^* melanoma cell subpopulation, characterised by reduced MHC‐I expression, enriched in advanced‐stage tumours and associated with worse survival and immunotherapy response. CRISPR screening further showed that *NARS2* and *NDUFC2* are necessary for the proliferation of melanoma cells, highlighting these genes as potential therapeutic targets. Tumour‐associated macrophages (TAMs) originate from both *FCN1* ^+^ monocytes and *FOLR2^+^* tissue‐resident macrophages, displaying two polarisation states with distinct prognostic associations. Specifically, pro‐inflammatory *CXCL9^+^CXCL10^+^* TAMs are enriched in SCC, while tissue‐remodelling *SPP1* ^+^ TAMs are predominant in melanoma. Immunofluorescence staining confirmed that *SPP1* ^+^ macrophage accumulation correlates with advanced stage, metastasis and poor prognosis in the melanoma cohort. Immune ecotype analysis reveals a transition from ‘T‐cell‐dominant’ ecotypes to ‘desert’ ecotypes as disease advances in BCC, CM and AM. Cell‒cell communication analysis shows that ‘T‐cell‐dominant’ ecotypes have higher MHC‐I signalling pathways in tumour cells, whereas ‘Desert’ ecotypes have higher *SPP1^+^* macrophage signalling, underlining the role of *SPP1* on immune remodelling. Functional assays confirm that melanoma cells could drive M2 polarisation and SPP1 upregulation in macrophages. Knocking down or overexpressing *SPP1* correspondingly alters M2 gene expression in macrophages.

**Conclusions:**

This study establishes a pan‐skin cancer immune remodelling framework, providing a foundation for biomarker discovery and the development of new immunotherapy strategies.

## Graphical abstract

Graphical highlights

Tumour subpopulations with high malignancy show reduced interferon signalling and reduced MHC‐I expression.

Tumour‐associated macrophages (TAMs) differentiate into two opposing states: SPP1^+^ TAMs and CXCL9^+^ TAMs.

Among immune ecotypes, T‐cell‐enriched ecotypes are linked with high MHC‐I expression in tumour cells while desert ecotypes are accompanied by high SPP1^+^ TAMs.

SPP1 regulates M2 markers in macrophages validated by functional assays.

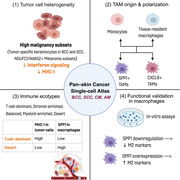

## 1 INTRODUCTION

Skin cancers include distinct subtypes: basal cell carcinoma (BCC), squamous cell carcinoma (SCC), cutaneous melanoma (CM) and acral melanoma (AM). Although these tumours share a cutaneous origin, they exhibit marked heterogeneity in clinical behaviours, metastatic potential and prognosis.^1^ BCC and SCC account for over 95% of skin cancer cases, and are predominantly localised and effectively treated by surgical excision. However, a rare fraction of the BCC and SCC patients progress to an advanced disease, requiring systemic therapy, such as targeted therapy or immunotherapy.^2^ In contrast, CM and AM are less common. However, they are aggressive and account for most skin cancer‐related deaths. Importantly, their response rates to immune checkpoint inhibitors differ substantially, with an approximately response rate of 40%‒60% in CM and only 20%‒30% in AM in advanced stage, underscoring the existence of subtype‐specific immune landscapes and resistance mechanisms.^3^, ^4^, ^5^

Tumour microenvironment (TME) comprises malignant and non‐malignant cellular and molecular components, such as immune cells, fibroblasts, cytokines and growth factors, that collectively shape tumour progression and therapeutic response. TME undergoes dynamic remodelling during disease evolution, characterised reprogramming of myeloid and lymphoid compartments, spatial reorganisation, and altered T‐cell functional states. Recent single‐cell RNA sequencing (scRNA‐seq) analyses have revealed that the TMEs of skin cancers possess distinct cellular and molecular features.^6^, ^7^ For example, CM forms a PD‐1/PD‐L1‐driven immunosuppressive boundary, which strengthens during progression, and AM has an ‘immune‐cold’ phenotype.^8^, ^9^, ^10^ BCC displays a more inhibitory TME in the infiltrative subtype compared to the nodular subtypes.^11^, ^12^ A recent pan‐skin cancer analysis of cancer‐associated fibroblasts (CAFs) revealed dynamic phenotype transitions that correlate with increasing tumour malignancy.^13^ However, no study has yet systematically integrated BCC, SCC, CM and AM to delineate their shared characteristics and distinct patterns of tumour progression and immune remodelling. Furthermore, the biological differences between aggressive malignancies such as melanoma and relatively indolent tumours such as BCC are of critical importance, yet remain incompletely understood.

To address the gap in understanding immune characteristics in skin cancers, we integrated 102 single‐cell samples from patients with BCC, SCC, CM and AM, encompassing adjacent normal skin as well as early‐ and advanced‐stage tumours. Using integrative scRNA‐seq analyses, we systematically characterised immune remodelling across skin cancer types and validated key findings using bulk RNA‐seq prognostic cohorts, immunofluorescence staining and in vitro assays. We identified a shared melanoma cell subpopulation characterised by MHC‐I downregulation and associated with poor patient survival, and uncovered divergent macrophage polarisation programs shaped by tumour context. Additionally, we identified five distinct immune ecotypes across skin cancers and revealed stage‐dependent transitions, highlighting *SPP1* ^+^ macrophages as key differential components among ecotypes. Together, these findings provide a comprehensive framework for understanding skin cancer immunobiology and highlight candidate targets for personalised immunotherapy.

## 2 METHODS

### 2.1 Patients and sample collection

This study was approved by the Ethics Committee of Central South University Xiangya Hospital (approval number: 202202043), and written informed consent was obtained from all participants. In‐house and public dataset information are illustrated in ‘Data Availability Statement’ section. Clinical staging was determined according to the AJCC 8th edition TNM classification and Brigham and Women's Hospital systems.^14^, ^15^ For BCC and SCC, tumours were staged following the criteria: lesions ≤2 cm without high‐risk pathological features (e.g., perineural invasion, deep invasion, poor differentiation) were defined as early stage, whereas tumours >2 cm or presenting ≥2 high‐risk features were considered advanced stage.^14^, ^15^ For some samples lacking complete clinicopathological data, tumour stage was obtained from the original study annotations. For CM and AM, stages I‒II (localised disease without lymph node or distant metastasis) were defined as early stage, and stages III‒IV (regional lymph node involvement or distant metastasis) as advanced stage.^14^

### 2.2 Immunofluorescence staining

Immunofluorescence staining was performed as previously described.^16^ In brief, the primary antibodies, including CD68 (1:100, Abcam mouse ab955) and SPP1 (1:100, Bio‐Techne, Goat AF1433) were incubated overnight at 4°C. Fluorescent second: Alexa Fluro 488 donkey anti‐goat (Invitrogen A11055) and Alexa Fluro 594 donkey anti‐mouse (Invitrogen A21203) incubation for 1 h at room temperature. PhenoImager HT was used to capture the images and identify all markers of interest. One to three representative fields of the whole‐slide scan images were selected and quantitatively analyzed by ImageJ. The staining and analysis results of the immunofluorescence staining were also checked by two certified pathologists.

### 2.3 Cell culture

The cell lines THP‐1, SK‐MEL‐28, RAW264.7 and B16 were obtained from the American Type Culture Collection. The human monocyte cell line THP‐1 was cultured in RPMI‐1640 medium (Gibco, C11875500BT) with 10% foetal bovine serum (FBS, ExCell, FSP500), 1% penicillin‒streptomycin (BI, 03‐031‐1B) and .05 mM β‐mercaptoethanol (Gibco, 21985‐023). To induce macrophage differentiation, THP‐1 cells were treated with 100 ng/mL phorbol 12‐myristate 13‐acetate (PMA; Sigma, P8139) for 24 h. Human melanoma cell line SK‐MEL‐28 and murine macrophage cell line RAW264.7 were maintained in DMEM medium (Gibco, C11995500BT) supplemented with 10% FBS. Murine melanoma cell line B16 cells were cultured in RPMI‐1640 medium containing 10% FBS.

### 2.4 Conditioned medium and co‐culture assay

For conditioned medium preparation, melanoma cell lines were cultured until 70%‒80% confluence, and conditioned medium was collected after 48 h, centrifuged to remove debris and stored at ‒80°C. Macrophage cell lines were then incubated in melanoma‐conditioned medium for the indicated time periods, while control macrophages cell lines were maintained in fresh complete DMEM.

### 2.5 Western blot assay

Western blot was performed as previously described.^17^ Whole‐cell lysates were lysed using RIPA lysis. Proteins were separated on a 10% SDS‒PAGE gel and identified by the following antibodies: SPP1 (1:1000 Abcam, ab63856) and α‐Tubulin Polyclonal Antibody (Proteintech 11224‐1‐AP).

### 2.6 Real‐time quantitative PCR

Total RNA was extracted using MagZol Reagent (Magen, R4801‐01), and cDNA was synthesised with Hifair II Reverse Transcriptase (Yeasen, 11141ES60). Quantitative real‐time PCR (qRT‐PCR) was performed using 2× SYBR Green qPCR Master Mix (bimake, with each sample run in triplicate. Gene expression levels were calculated by normalizing the threshold cycle (Ct) values of the target genes to that of β‐actin. All primer sequences are shown in Table S5.

### 2.7 Short hairpin RNA knockdown and SPP1 overexpression

For short hairpin RNA (shRNA) knockdown and overexpression, HEK293T cells were transfected with lentiviral plasmid, psPAX2 and pMD2.G by lipofectamine 2000 transfection reagent (Thermo Fisher 11668019). The supernatants were collected and filtered 48 h after transfection, and then the target cell lines were infected. Puromycin was added to obtain stable cell lines with successful transduction after 48 h. The sequences of the shRNAs were as follows: shSPP1 1# (homo): GCTACAGACGAGGACATCA; shSPP1 2# (homo): CCGTGGGAAGGACAGTTAT.

### 2.8 Preparation of single‐cell suspensions

Fresh tissue samples were immersed in MACS Tissue Storage Solution (Miltenyi Biotec, 130‐100‐008) on ice within 30 min post‐surgery. Prior to dissociation, the samples were rinsed three times using Hanks’ balanced salt solution. To generate single‐cell suspensions, we utilised the Human Tumour Dissociation Kit (Miltenyi Biotec) in combination with the gentle MACS Dissociator (Miltenyi Biotec) and gentleMACS C tubes (Miltenyi Biotec). To enhance cell viability for downstream scRNA‐seq, dead cells were removed using the Dead Cell Removal Kit (Miltenyi Biotec).

### 2.9 Single‐cell RNA sequencing and data preprocessing

Single‐cell transcriptome libraries were generated using the BD Rhapsody, 10× Genomics Chromium, and Singleron GEXSCOPE platforms as previously described.^16^

For the BD Rhapsody system, single cells were barcoded with the Human Immune Single‐Cell Multiplexing Kit (BD Biosciences) and processed following the manufacturer's protocol. Libraries were sequenced on an Illumina NovaSeq 6000 platform (2 × 100 bp, >200 000 reads per cell) with 20% PhiX spike‐in to enhance base diversity.

For the 10× Genomics platform, single‐cell suspensions were loaded into the Chromium Controller, and libraries were prepared using the Chromium Single Cell 5′ Library & Gel Bead Kit (10× Genomics) and sequenced on the Illumina HiSeq X Ten platform (150 bp paired‐end).

For the Singleron platform, cells were processed using the Matrix Single Cell Processing System and GEXSCOPE Single Cell RNA Library Kits (Singleron), followed by sequencing on the Illumina HiSeq X platform (150 bp paired‐end).

Raw sequencing data from our in‐house and six publicly available datasets (Table S1) were uniformly processed. Data from 10× Genomics, BD Rhapsody and Singleron were analysed using the CellRanger (V6.1.2), BD Rhapsody Analysis Pipeline and scopetools workflows, respectively. All reads were aligned to the GRCh38 (Ensembl V99) reference genome. For external datasets providing only normalised expression matrices (CPM/TPM), those matrices were directly used for downstream analyses.

### 2.10 Quality control of scRNA‐seq data

For both in‐house and public data, we applied strict quality control rules. Cell quality was evaluated using multiple metrics: (i) total unique molecular identifiers (UMIs) per cell, (ii) the number of genes per cell, (iii) the number of housekeeping genes expressed per cell, and (iv) the fractions of mitochondrial transcripts. Cells with gene number <200 were removed. In addition, cells with UMIs, total gene number or housekeeping gene count below the median minus 3 × median absolute deviation within each sample were excluded.^18^ Cells with mitochondrial gene proportions greater than 20% were removed. Potential doublets were identified and excluded using Scrublet with default settings.^19^

### 2.11 scRNA‐seq data integration and major cell type annotation

The Seurat (V4.4.0) pipeline was used for standardisation, dimensionality reduction and visualisation of single‐cell sequencing data.^20^ Gene expression values were normalised using the ‘LogNormalise’ approach with a scaling factor of 10 000. Identification of highly variable genes and data scaling were carried out through the ‘FindVariableFeatures’ and ‘ScaleData’ functions, respectively. Principal component analysis (PCA) was subsequently applied for dimensionality reduction via the ‘runPCA’ function, followed by uniform manifold approximation and projection (UMAP) to compute the low‐dimensional embedding. To correct for batch effects and enable cross‐sample integration across platforms, the Harmony algorithm was utilised.^21^ For the annotation of major cell subtypes, we used the ‘FindClusters’ function and the ‘FindAllMarkers’ function separately to identify cell clusters and differentially expressed genes. Cell populations were primarily annotated based on the expression of classical markers: melanocytes (*PMEL*, *MLANA* and *MITF*), epithelial cells (*KRT17*, *KRT14* and *EPCAM*), T/natural killer (NK) cells (*CD2* and *CD3D*), endothelial cells (*VWF* and *PECAM1*), fibroblasts (*DCN* and *COL1A1*), monocytes/macrophages cells (*LYZ* and *CD14*), dendritic cells (DCs) (*LYZ*) and B/plasma cells (*JCHAIN* and *CD79A*).

### 2.12 Pearson residual calculation in contingency tables

The enrichment or depletion of specific cell clusters across groups was assessed using Pearson residuals, which quantify the deviation of observed cell counts from expected values in a contingency table. The residual was calculated as: *R* = (obs − exp)/√exp, where ‘obs’ is the observed number of cells and ‘exp’ is the expected number, determined by multiplying the marginal probabilities of the row and column and scaling by the total number of cells. Positive residuals indicate relative enrichment, whereas negative residuals denote depletion.^22^

### 2.13 Copy number variation inference and malignant cell identification

Copy number variations (CNVs) were inferred using InferCNV (V1.16.0; https://github.com/broadinstitute/infercnv) to identify malignant populations from epithelial or melanocytic lineages, with T and NK cells serving as internal reference controls for each tumour sample. This analysis encompassed melanocytes from CM and AM and epithelial cells from BCC and SCC. For samples exhibiting weak CNV signals, malignant identity was further validated by assessing the expression of canonical tumour markers.

### 2.14 Trajectory inference of monocytes and macrophages

To infer the evolutionary dynamics of monocytes and macrophages, we applied a combination of computational approaches. Initially, CytoTRACE was employed to estimate the relative differentiation potential (stemness) across cell clusters.^23^ Subsequently, trajectory inference was performed using Monocle3, which allowed us to reconstruct lineage relationships among subpopulations.^24^ The root of the trajectory was defined based on a combination of known marker gene expression, tissue localisation, and CytoTRACE‐derived stemness scores, enabling the delineation of a developmental path.

### 2.15 Differentially expressed genes and gene set enrichment analysis

To identify the differentially expressed genes across cell types, we used the FindMarkers function in Seurat. Marker genes for the cell cluster of interest were defined using a threshold of log_2_ fold change >.4 and adjusted *p*‐value <.05. To investigate the functional characteristics of specific cell subtypes, gene set enrichment analysis was conducted based on the DEGs using the clusterProfiler package (V4.1.4).^25^ Pathways with a *p* < .05 were considered significantly enriched.

### 2.16 Calculation of function module scores

To evaluate specific functional modules, we computed module scores for individual cells using the ‘AddModuleScore’ function in Seurat. Signature gene sets were curated from the Hallmark and Gene Ontology databases, as well as previously published functional signatures, including immunosuppression of myeloid cells, cytotoxicity and exhaustion/inhibitory functions of T cells. The immunosuppression score was calculated using the previous reported gene signature of human myeloid‐derived suppressor cells, excluding cellular marker genes.^26^ The cytotoxicity score was determined using the gene list of *PRF1*, *IFNG*, *GNLY*, *NKG7*, *GZMA*, *GZMH*, *KLRK1*, *KLRB1*, *KLRD1*, *CTSW* and *CST7*.^27^ The exhausted/inhibitory score was defined using the gene list of *CXCL13*, *HAVCR2*, *PDCD1*, *TIGIT*, *LAG3*, *CTLA4*, *LAYN*, *RBPJ*, *VCAM1*, *TOX* and *MYO7A*.^27^

### 2.17 Identification of skin cancers ecotypes

To investigate how different cellular compartments and subsets in the skin cancer microenvironment form cohesive ecosystems, we conducted an unsupervised analysis to infer intercellular relationships and co‐association patterns. First, we quantified the cellular composition by calculating the fractions of 44 distinct cell populations across 102 skin cancer samples. Next, we derived the relative abundance of each cell subset by scaling the sample‐by‐cell‐type proportion matrix across cell types or states. Finally, unsupervised hierarchical clustering was performed on the scaled matrix using the pheatmap R package, with the parameter clustering_method = ‘ward.D2’, to infer co‐existence patterns of cell populations across samples. This analytical framework was adapted from a previously published study on gastric cancer.^28^

### 2.18 Cell‒cell interaction analysis

Cellular interaction events between the interested cell types were investigated using the CellChat (V1.1.3) method.^29^ In summary, we adhered to the official workflow, importing the normalised counts into CellChat and then executed the preprocessing functions ‘identifyOverExpressedGenes’, ‘identifyOverExpressedInteractions’ and ‘projectData’ with default parameters. Interaction networks were visualised using the ‘netVisual_aggregate’ function.

### 2.19 Estimation of cell subtype enrichment in bulk RNA‐seq data based on scRNA‐seq signatures

To estimate the enrichment of scRNA‐seq‐derived cell subpopulations in bulk RNA‐seq data, we first identified subpopulation‐specific signature genes from the single‐cell dataset. Differentially expressed genes were determined for each cluster within its lineage. To improve specificity, genes that were also highly expressed in other major cell lineages were excluded, resulting in a refined set of subpopulation‐specific signature genes (Mono/Macro_c2: *INHBA*, *CCL20*, *IL1RN*, *SLC7A11*, *CXCL3*, *VEGFA*; Mono/Macro_c4: SPP1, TREM2; Mono/Macro_c5/c6: *CXCL9*, *CXCL10*, *ANKRD22*; Mono/Macro_c8/c9: *FOLR2*, *SELENOP*, *SLC40A1*). For deconvolution of bulk RNA‐seq data, we applied the ‘ssgsea’ function from the GSVA R package (V1.44.5) to calculate enrichment scores for each sample. These scores represent the relative enrichment of gene signatures corresponding to specific cell subpopulations.

### 2.20 Survival analysis

We collected bulk RNA‐seq data and clinical information from the GSE19234 melanoma dataset. Kaplan‒Meier survival curves were generated using the survival R package (V2.4) and visualised with survminer (V0.4.9). Optimal cutoffs for dividing samples into high and low expression/signature score groups were determined automatically using the ‘surv_cutpoint’ function. Survival differences were evaluated using the log‐rank test.

### 2.21 Gene dependency analysis using CRISPR screening data

To evaluate the functional importance of target genes in melanoma, we queried CRISPR‐Cas9 gene dependency data from the ICRAFT (https://icraft.pku‐genomics.org/#/homepage) and DepMap portal (https://depmap.org), specifically the DepMap CRISPR (Avana) Public 22Q4 dataset. This dataset provides genome‐wide loss‐of‐function screening data across hundreds of cancer cell lines. We focused our analysis on melanoma cell lines. Gene effect scores were retrieved for each target gene, where a lower score (typically < ‒.5) indicates a higher dependency of the cell line on the gene for survival in DepMap.

### 2.22 Statistical analysis

All statistical analyses were performed in R (V4.3.1) and GraphPad Prism (V9.5.1). Depending on the data type and comparison, appropriate statistical tests were applied, including the Wilcoxon rank‐sum test, Kruskal‒Wallis's test, *t* test, one‐way ANOVA and Spearman's correlation analysis. Overall survival was evaluated using the log‐rank test.

## 3 RESULTS

### 3.1 Single‐cell atlas of skin tumours

**FIGURE 1:**
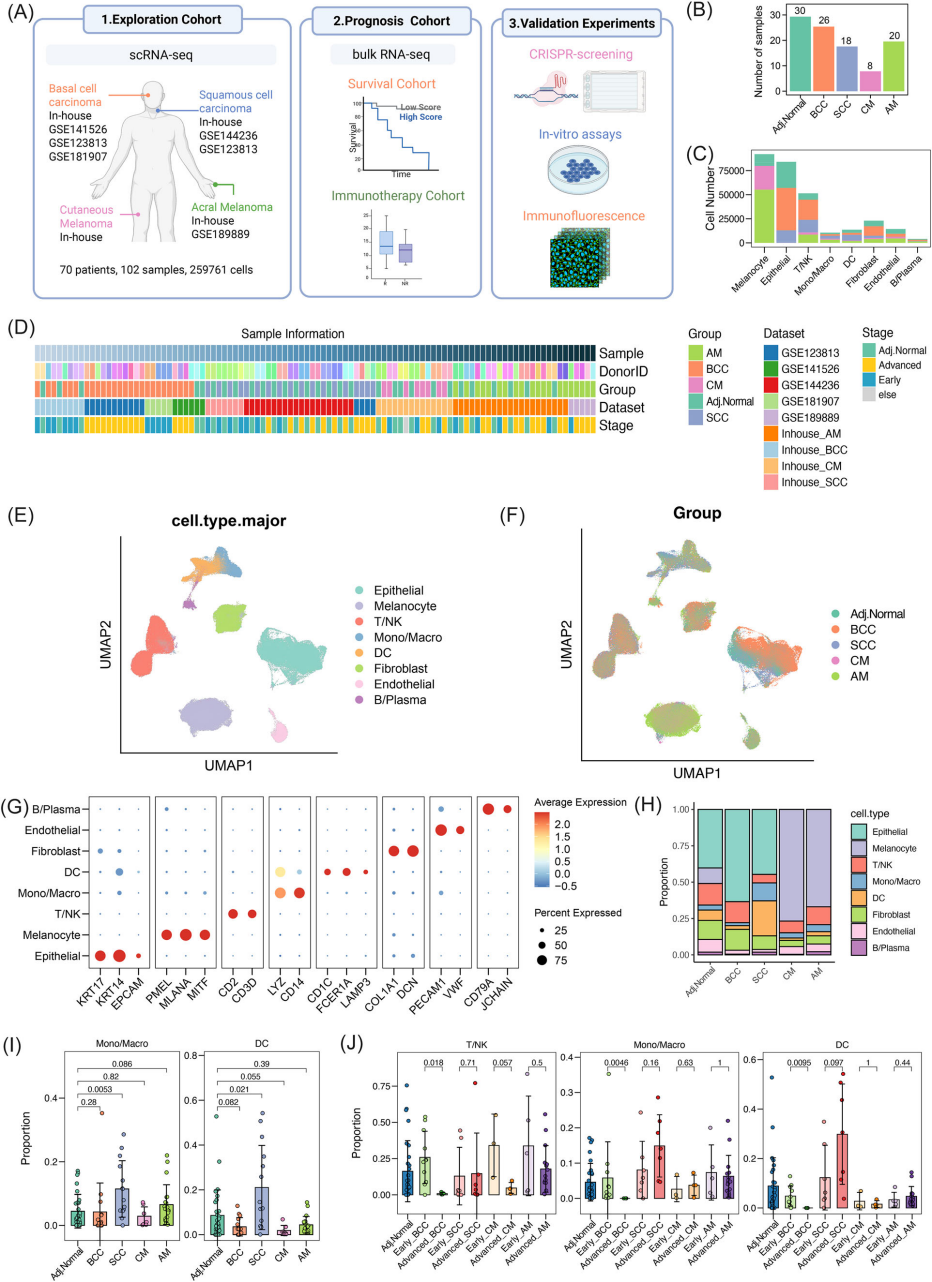
Study design for generating a single‐cell atlas of skin tumours. (A) Schematic overview of the study design. (B) Bar chart showing the number of samples across groups. (C) Bar chart showing the number of cells per major lineage. (D) Overview of sample metadata. (E) Uniform manifold approximation and projection (UMAP) visualisation of major cell lineages across all samples. (F) UMAP visualisation of all cells coloured by groups. (G) Dot plot showing canonical marker expression in major cell lineages. (H) Stacked bar plots of lineage composition across tumour groups. (I and J) Bar plots of indicated immune cell lineage proportions across tumour types (I) and stages (J). p‐Values were calculated using the two‐sided Wilcoxon rank‐sum test. Data are presented as mean ± SD. Adj. Normal, adjacent normal skin; AM, acral melanoma; BCC, basal cell carcinoma; CM, cutaneous carcinoma; DC, dendritic cells; SCC, squamous cell carcinoma.

We built a comprehensive scRNA‐seq atlas of skin cancers by integrating in‐house‐generated and publicly available scRNA datasets (Figure S1A and Table S1). The cohort included early‐ and advanced‐stage tumours and adjacent normal skin samples from four major skin cancer types (Figure 1A‒C). In total, 102 single‐cell samples were obtained from 70 patients, including 26 BCC, 18 SCC, eight CM, 20 AM tumour samples and 30 adjacent normal skin samples. Among them, 51 samples were generated by our group (47 previously published and four newly generated),^7^, ^11^, ^16^, ^17^ and the remaining 51 samples were obtained from five public datasets.^6^, ^9^, ^30^, ^31^, ^32^ We also collected available clinical metadata, such as sex, age and tumour stage (early vs. advanced), which was based on the AJCC 8th edition TNM system and the Brigham and Women's Hospital risk classification system (Table S2 and Section 2).^14^, ^15^

After stringent quality control and filtering, a total of 25 9761 high‐quality cells were obtained for analysis. Samples from different datasets were integrated using the standard Seurat workflow, followed by batch effect correction with Harmony (Figure 1E,F). Comparison of UMAP plots before and after correction demonstrated effective batch correction among different technology platforms (Figure S1B). Cell types were annotated based on cell‐type‐specific gene markers, forming eight major cell lineages, including melanocytes, epithelial cells, T/NK cells, monocytes/macrophages (Mono/Macro), DCs, fibroblasts, endothelial cells and B/plasma cells (Figure 1G). Comparisons of major cell lineages across cancer types and stages revealed distinct tumour‐type‐specific patterns. SCC displayed the highest abundance of Mono/Macro and DC populations among tumour types (Figure 1H,I). Stage‐specific comparisons further demonstrated a notable reduction of T/NK cells in advanced BCC and CM compared to their early‐stage counterparts. Moreover, advanced BCC exhibited decreased proportions of Mono/Macro and DC cells compared to the early stage (Figure 1J). These results indicate the existence of heterogeneous immune remodelling processes during skin tumour progression, and we therefore examined in‐depth to characterise the landscapes of immune cells, cancer cells, ecotypes, as well as cell‒cell communications.

### 3.2 Melanoma cell subsets possess stage‐associated malignant features and MHC‐I linked immune evasion

**FIGURE 2:**
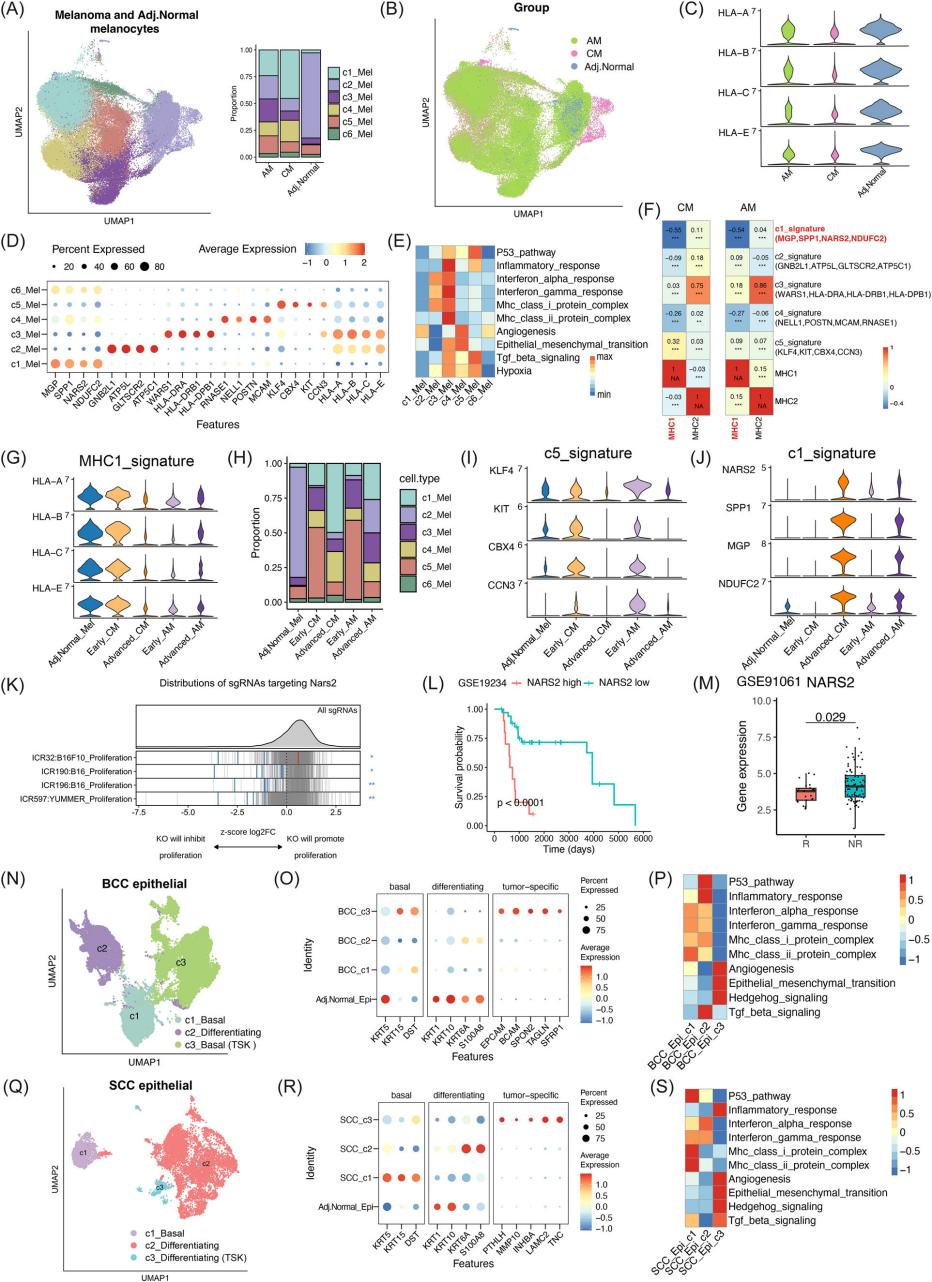
Landscape of tumour cells across skin tumour types and stages. (A) Uniform manifold approximation and projection (UMAP) of melanoma and adjacent normal skin melanocytes (Mel) coloured by clusters, with a bar chart showing the cluster distribution across groups. (B) UMAP plots of coloured adjacent normal skin, cutaneous carcinoma (CM) and acral melanoma (AM) groups. (C) Violin plots of MHC class I (HLA) expression across groups. (D) Dot plot of differentially expressed genes across Mel subsets. (E) Heatmap showing hallmark pathway activity across Mel subsets. (F) Heatmap of Spearman correlation between Mel subsets signatures and MHC signatures. (G) Violin plots of HLA expression across groups. (H) Proportions of Mel subsets across groups. (I and J) Violin plots showing gene expression of c5_Mel (I) and c1_Mel (J) signatures across groups. (K) Distribution of sgRNAs targeting *NARS2* from ICRAFT CRISPR screens. The *X*‐axis indicates the effect of NARS2 knockout on cell proliferation. Negative values (left) mean proliferation is inhibited, while positive values (right) mean proliferation is promoted. Each line represents an sgRNA; the grey density curve shows the overall distribution. Blue lines/asterisks mark significantly depleted guides (KO inhibits proliferation), and the red line indicates enriched guides. (L) Kaplan‒Meier survival analysis stratified by *NARS2* expression using the bulk‐RNA melanoma cohort (GSE19234, *n* = 44). (M) *NARS2* expression in immunotherapy responders (R) versus non‐responders (NR) of the bulk‐RNA melanoma cohort. p‐Values were calculated using the two‐sided Wilcoxon rank‐sum test (GSE91061, *n* = 105, R = 23, NR = 82). (N) UMAP plot of epithelial subclusters in basal cell carcinoma (BCC). (O) Dot plots showing marker expression across BCC subclusters. (P) Heatmap showing hallmark scores of BCC subclusters. (Q) UMAP plot of epithelial subclusters in squamous cell carcinoma (SCC). (R) Dot plots showing marker expression across SCC subclusters. (S) Heatmap showing hallmark scores of SCC subclusters. ^*^ *p* < .05, ^**^ *p* < .01, ^***^ *p* < .001, ^****^ *p* < .0001. HLA, human leukocyte antigen; sgRNA, single‐guide RNA; KO, knockout.

Inter‐ and intratumoural heterogeneity define tumour identity and influence prognosis and treatment response. To dissect the tumour cell compartment, we characterised transcriptional features of malignant subpopulations within each cancer type and also compared the overall features across cancer types. Using InferCNV, we inferred CNVs to identify malignant cells from epithelial or melanocytic lineages, with T/NK cells serving as internal references for each tumour sample. This analysis encompassed melanocytes from CM and AM, as well as epithelial cells from BCC and SCC (Figure S2A). For samples with low CNV patterns, we also assessed canonical tumour markers expression. We next integrated melanocytes from adjacent normal skin samples and melanoma cells from CM and AM tumour samples, and identified six transcriptionally distinct subpopulations (Figure 2A,B). Since MHC‐I expression (e.g., HLA‐A) is central to immune recognition,^33^ we compared HLA levels across groups. Adjacent normal skin melanocytes displayed higher HLA expression, whereas melanoma cells from CM and AM showed markedly reduced levels, suggesting immune evasion (Figure 2C).

To further characterise the molecular features underlying these differences, We defined gene expression signature for each cluster based on their highly expressed genes: c1_Mel (*MGP*, *NARS2*, *SPP1*, *NDUFC2^+^*), c2_Mel (*GNB2L1*, *ATP5L*, *GLTSCR2*, *ATP5C1* ^+^), c3_Mel (*WARS1*, *HLA‐DRA*, *HLA‐DRB1*, *HLA‐DPB1* ^+^), c4_Mel (*RNASE1*, *NELL1*, *POSTN*, *MCAM* ^+^), c5_Mel (*KLF4*, *CBX4*, *KIT*, *CCN3* ^+^) and c6_Mel that shares marker genes with c1_Mel (Figure 2D and Table S3). Further hallmark pathway scoring revealed functional diversity among these subsets. c1_Mel displayed low ‘immune activity’ but high ‘angiogenesis’ scores. c2_Mel and c3_Mel were enriched for ‘interferon response’ and ‘MHC‐I protein complex’, indicating enhanced immune signalling potential. c4_Mel showed concurrent enrichment in the ‘angiogenesis’ and ‘epithelial‒mesenchymal transition (EMT)’ pathways, accompanied by reduced immune functions. c5_Mel was enriched for ‘P53 pathway’ and ‘TGF‐β signalling’, suggesting a potential tumour‐suppressive activity (Figure 2D,E). These findings suggest that each melanoma comprises specialised subsets with distinct functional programs.

To further link these transcriptional states to immune modulation, we performed correlation analysis between subset signatures and MHC‐I expression. The c1_Mel signature showed a significant negative correlation with MHC‐I expression in both CM and AM (CM: *r* = ‒.55; AM: *r* = ‒.54; both *p* < .001), consistent with immune‐evasive function. Similarly, c4_Mel showed a consistent negative trend with MHC‐I expression (CM: *r* = ‒.26; AM: *r* = ‒.27; both *p* < .001). In contrast, c5_Mel showed a positive correlation with MHC‐I in CM (*r* = .32, *p* < .001) but not in AM (*r* = .09, *p* < .001), suggesting subtype‐specific immune interactions (Figure 2F). This pattern highlights immune‐inert subsets and AM‐specific constitutive immune evasion mechanisms.

To assess whether MHC‐I differences are associated with disease stage, we examined MHC‐I expression across tumour stages. Early‐stage CM cells resembled adjacent normal skin melanocytes, showing high MHC‐I levels, whereas advanced CM showed pronounced downregulation. In AM, however, both early‐ and advanced‐stage tumours exhibited persistently low MHC‐I expression, suggesting that immune evasion is established earlier and maintained more robustly in AM (Figure 2G). Mechanistically, this potentially explained the poor immunotherapy response in AM compared to CM. We next examined whether the composition of melanoma subsets varies across disease stages. The c5_Mel and its signature genes (*KLF4*, *CBX4*, *KIT*, *CCN3* ^+^) were relatively enriched in early‐stage CM and AM, whereas the c1_Mel and its signature genes (*MGP*, *NARS2*, *SPP1*, *NDUFC2* ^+^) were more abundant in advanced‐stage CM and AM (Figure 2H‒J). To further evaluate the clinical relevance of melanoma subpopulations, we performed survival analyses based on the signature scores of representative melanoma subsets, including *NARS2/NDUFC2* ^+^c1_Mel, c5_Mel and c2_Mel. Notably, we found that a high c1_Mel signature score was significantly associated with worse survival (*p* = .00062), whereas a high c2_Mel signature score showed a trend towards improved survival outcomes (*p* = .057), corresponding with its high enrichment feature in adjacent normal skin. Interestingly, the c5_Mel signature did not show a significant association with patient survival (*p* = .32). Collectively, these results underscore the intratumoural heterogeneity of melanoma and the clinical importance of the *NARS2/NDUFC2* ^+^c1_Mel subpopulation (Figure S2C‒E).

Next, we evaluated DepMap CRISPR‒Cas9 loss‐of‐function screening data for genes comprising the c1_Mel and c5_Mel signatures. Notably, NDUFC2 and NARS2 consistently showed among the most negative CRISPR gene effect scores across both CM and AM cell lines, indicating that loss of these genes markedly impaired cell proliferation (Figure S2F). Inquiring in iCRAFT database further supported that knockouts of *NARS2* significantly impaired proliferation in multiple melanoma cell lines (Figure 2K). NDUFC2 encodes a subunit of mitochondrial respiratory chain complex I,^34^ while NARS2 encodes an aminoacyl‐tRNA synthetase, essential for mitochondrial protein synthesis.^35^ Functional enrichment analyses revealed that the c1_Mel subpopulation was characterised by elevated oxidative phosphorylation, ATP synthesis and mitochondrial‐related functions, indicating a high‐energy metabolic state (Figure S2B). Clinically, analysis of bulk RNA‐seq melanoma cohorts demonstrated that high *NARS2* expression was associated with worse survival (*p* < .001; Figure 2L). Moreover, in immunotherapy datasets, *NARS2* was elevated in non‐responders compared to responders (*p* = .029; Figure 2M). Together, these findings suggest that the c1_Mel signature, particularly *NARS2*, may serve as a potential prognostic and immunotherapy predictive marker in melanoma.

In BCC and SCC, tumour cells exhibited a conserved architecture comprising three main subsets: c1_basal cells (*KRT15*, *DST* ^+^), c2_differentiated cells (*KRT6A, S100A8* ^+^) and c3_tumour‐specific keratinocytes (TSKs; *EPCAM*, *BCAM*, *SPON2* ^+^ in BCC; *PTHLH*, *MMP10*, *LAMC2* ^+^ in SCC) (Figure 2N–S). Functional scoring revealed that c3_TSK cells displayed the lowest activities in ‘interferon signalling’, ‘MHC‐I protein complex’ and ‘P53 pathway’ while exhibiting the highest ‘angiogenesis’ and ‘EMT’ scores in both BCC and SCC (Figure 2P,S), consistent with a pro‐invasive but immune‐silent phenotype. Comparative profiling across BCC, SCC, CM and AM further revealed cancer type‐ and stage‐specific programs: BCC and SCC were dominated by strong ‘P53 pathway’ activation; SCC and early‐stage CM exhibited high ‘interferon signalling’ and ‘MHC protein complex’ activities; in contrast, advanced CM and both early‐ and advanced‐stage AM displayed elevated angiogenesis and EMT scores (Figure S2G), underscoring progressive acquisition of invasive and immune‐evasive phenotypes in melanoma.

Collectively, these findings delineate melanoma subpopulations by revealing their distinct characteristics, including their unique transcriptional programs, stage‐specific dynamics and MHC‐I‐associated immune evasion. In contrast, BCC and SCC exhibited conserved epithelial architectures. These insights deepen our understanding of skin cancer heterogeneity, providing potential prognostic and therapeutic targets.

### 3.3 Monocyte and macrophage landscape in skin tumours

**FIGURE 3:**
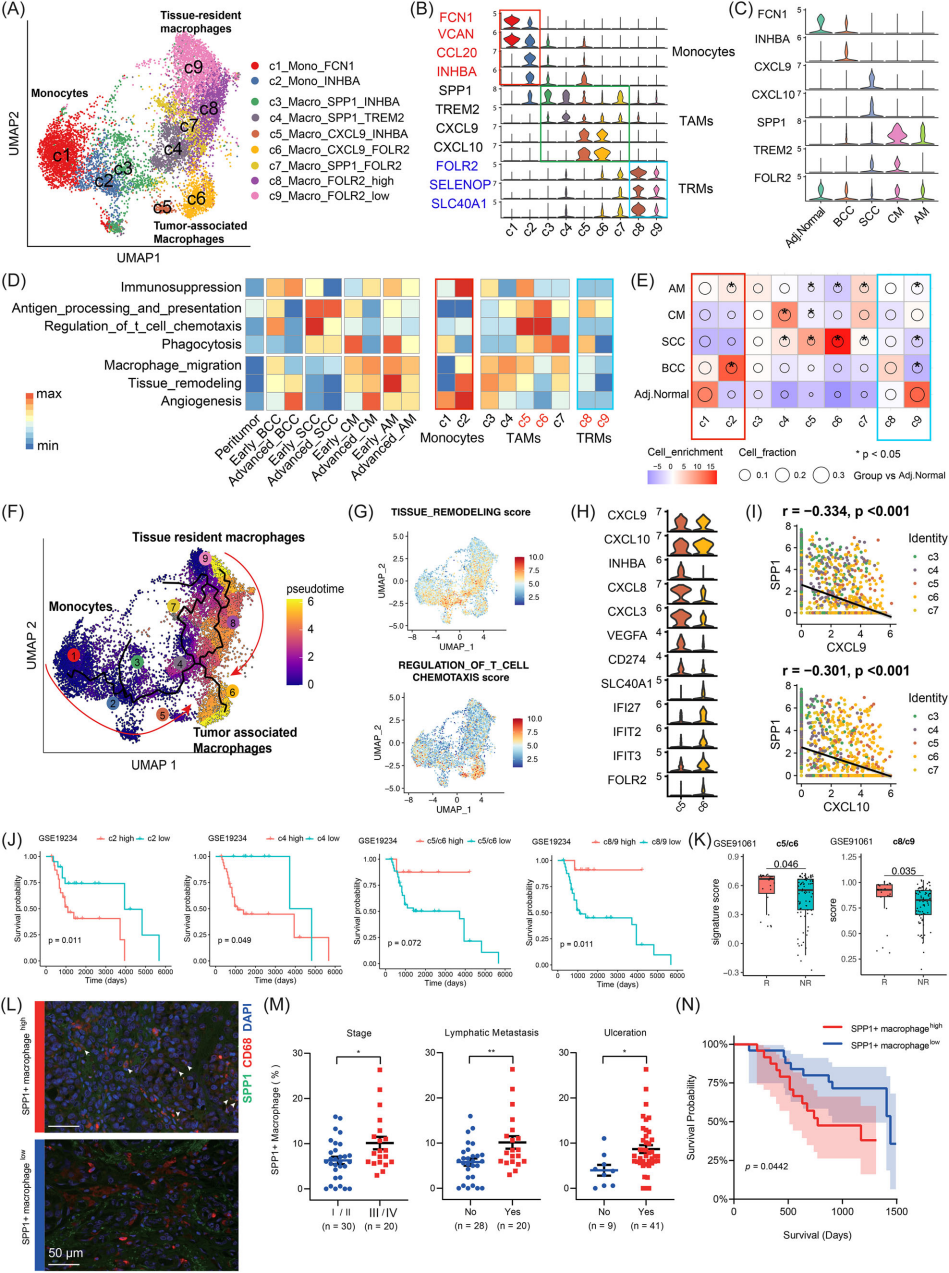
Landscape of monocytes and macrophages in skin tumours. (A) Uniform manifold approximation and projection (UMAP) plot of monocyte/macrophage subsets. (B) Violin plots of representative marker genes for monocyte/macrophage subsets. (C) Violin plots of marker gene expression across groups. (D) Heatmaps showing pathway scores across skin tumour groups and stages (left), and across monocyte/macrophage subsets (right). (E) Distribution enrichment analysis of monocyte/macrophage subsets across groups. The size of the circle indicates the relative proportion of cell subsets, while colour shows the enrichment level (Pearson residual), and an asterisk denotes a significant change when compared to adjacent normal skin (*p* < .05, two‐sided Wilcoxon rank‐sum test). (F) Pseudotime trajectory of monocyte/macrophage subsets. (G) Functional module scores projected onto UMAP. (H) Violin plots of marker genes and differentially expressed genes of c5 and c6. (I) Scatter plots showing gene expression associations in tumour‐associated macrophage (TAM) subsets in tumour samples. p‐Values were calculated using spearman's correlation analysis. (J) Kaplan‒Meier survival curves stratified by gene signatures of macrophage subsets using the bulk‐RNA melanoma cohort (GSE19234, *n* = 44). (K) Expression of subset‐specific signatures in immunotherapy responders (R) versus non‐responders (NR) of the bulk‐RNA melanoma cohort (GSE91061, *n* = 105, R = 23, NR = 82). (L) Representative staining of *SPP1* and *CD68* in tissues from an in‐house melanoma immunofluorescence cohort (*n* = 50). Scale bars, 50 µm. (M) *SPP1* ^+^ macrophage proportion in melanoma tissues stratified by stage and pathological characteristics. p‐Values were calculated using the unpaired two‐tailed *t*‐test; ^*^ *p* < .05, ^**^ *p* < .01. (N) Survival analysis in the melanoma cohort based on *SPP1* ^+^ macrophage proportion. TRMs, tissue‐resident macrophages.

Next, we analysed myeloid populations across skin tumours. Unsupervised clustering of myeloid population revealed nine monocyte/macrophage subsets (Figures 3A and S3A,B): two monocyte subsets (c1: FCN1, VCAN^+^; c2: INHBA, CCL20^+^), two tissue‐resident macrophages (c8 and c9: FOLR2, SELENOP^+^) and five tumour‐associated macrophages (TAMs; c3‒c7). TAMs were further divided into pro‐inflammatory subsets (c5 and c6: CXCL9, CXCL10^+^) and tissue‐remodelling subsets (c3, c4 and c7: SPP1, TREM2^+^) (Figure 3B). Notably, SPP1 expression within the Mono/Macro compartment was the highest in CM and second highest in AM (Figure 3B,C). Functional module scoring further revealed tumour‐specific activity patterns. Mono/Macro in early‐stage BCC and SCC were enriched for ‘regulation of T‐cell chemotaxis’, whereas Mono/Macro in CM and AM exhibited low activity for this program. Conversely, CM and AM consistently showed high levels of ‘tissue remodelling’ and ‘angiogenesis’, underscoring the influence of tumour‐intrinsic factors in shaping Mono/Macro functions (Figure 3D).

Immunosuppression is known for its essential role in tumour evasion. Subset‐level analysis indicated that c2_Mono_INHBA as the most immunosuppressive population, with the highest ‘immunosuppression’ and ‘angiogenesis’ scores (Figure S3C). c2_Mono_INHBA, defined by high INHBA expression (a member of the TGF‐β family), was enriched in BCC and AM compared to adjacent normal skin group.^12^ Differential expression analysis revealed that c2_Mono_INHBA upregulated M2‐associated gene expression (e.g., *MRC1* and *CD163*)^36^ in both CM and AM groups (Figure S3D). Further functional enrichment analysis uncovered tumour‐specific programs: in BCC, ‘TGF‐β activation’ and ‘fibroblast proliferation’; in AM, ‘vasculature development’ and ‘immune inhibitory signalling’; but in SCC, ‘antigen presentation via MHC II’ and ‘regulation of B‐cell differentiation’ (Figure S3E). These findings indicate that c2_Mono_INHBA plays a central role in driving immunosuppression and angiogenesis in BCC, CM and AM, while having a relatively different immune‐response role in SCC. Collectively, these findings define a diverse Mono/Macro landscape across skin tumours and identify c2_Mono_INHBA as a key subset mediating tumour‐promoting immunosuppressive programs.

### 3.4 TAMs have distinct origins and polarisation trajectories in skin tumours

To trace the origins of TAMs in skin tumours, we analysed the Mono/Macro subsets across tumour contexts. We found that c1_Mono_FCN1 and c9_Macro_FOLR2_low were significantly enriched in the adjacent normal skin compartment and exhibited high stemness scores inferred by SCNET, and high differentiation potency scores estimated by Cytotrace2 (Figure S3F). Consistently, Monocle3 pseudotime trajectory analysis positioned both clusters at early stages of the Mono/Macro trajectory (Figure 3F), supporting their roles as precursor states. Consistently, c1_Mono_FCN1 expressed high levels of *FCN1* and *VCAN*, established markers of circulating monocytes,^37^ whereas c9_Macro_FOLR2_low showed high expression of *FOLR2* and *SELENOP*, which have been reported as markers of tissue‐resident macrophages (Figure 3B).^38^ Together, these findings indicate that TAMs can arise from both monocytes and tissue‐resident macrophages, with c1_Mono_FCN1 and c9_Macro_FOLR2_low serving as early precursors during tumour development.

The inferred trajectory revealed distinct differentiation directions. Along the monocyte‐derived trajectory (c1 → c2), there was a progressive increase in ‘immunosuppression’ signatures, whereas the tissue‐resident macrophages‐derived trajectory (c9 → c8) showed enhanced immune‐activating features, including ‘phagocytosis’ and ‘antigen presentation’ (Figure 3D). Notably, during tumour progression from early to advanced BCC, the relative proportions of monocytes (c1_Mono_FCN1) increased, whereas tissue‐resident macrophages (c8/c9_Macro_FOLR2) decreased, highlighting dynamic shifts in these origins (Figure S3G). This pattern likely reflects the stronger immunosuppressive programming of recruited monocytes, while tissue‐resident macrophages tend to maintain immune‐activating properties.

At the trajectory termini, TAMs showed two major functional states: (1) tissue‐remodelling/migratory TAMs (c3, c4, c7: *SPP1^+^*), which were enriched in CM, AM and SCC, but largely absent in BCC. (2) T‐cell‐recruiting/immune‐activating TAMs (c5, c6: *CXCL9*, *CXCL10^+^*), which were most abundant in SCC (Figure 3D–G). Despite shared expression of CXCL9/CXCL10, c5_Macro_CXCL9_INHBA displayed co‐expression of immunosuppressive genes (INHBA, CD274) and elevated ‘immunosuppression’ scores, while c6_Macro_CXCL9_FOLR2 was enriched for interferon‐associated genes (IFI27, IFIT2) (Figure 3H). Correlation analysis between SPP1 and CXCL9/CXCL10 expression in TAMs revealed negative associations (CXCL9: *r* = ‒.33; CXCL10: *r* = ‒.30; both *p* < .001), suggesting functional antagonism between tissue‐remodelling and T‐cell‐recruiting TAM subsets (Figure 3I).

To access the clinical relevance of these macrophage states, we performed survival and immunotherapy‐response analyses in the bulk‐RNA melanoma cohorts. High expressions of c2_Mono_INHBA signature (*INHBA*, *CCL20*, *IL1RN*, etc.) and c4_Macro_*SPP1*_TREM2 signature (*SPP1*, *TREM2*) were associated with poorer survival prognosis. In contrast, elevated expression of c5/c6 signature (*CXCL9*, *CXCL10*, *ANKRD22*) and c8/c9 signature (*FOLR2*, *SELENOP*, *SLC40A1*) correlated with improved survival prognosis (Figures 3J and S3H). Furthermore, in bulk‐RNA melanoma datasets with immunotherapy‐response information, c5/c6 and c8/c9 signature scores were significantly higher in responders compared to non‐responders (c5/c6: *p *= .046; c8/c9: *p *= .035) (Figure 3K).

To further validate the clinical significance of *SPP1* ^+^ macrophages, we established an in‐house cohort of 50 melanoma patients (49 acral and one cutaneous) and performed immunofluorescence staining to quantify *SPP1* ^+^ macrophages (Table S4). Patients were stratified into high and low groups based on the proportion of *SPP1* ^+^ macrophages (Figure 3L). The high infiltration of *SPP1* ^+^ macrophages closely linked to poor prognostic features including advanced stage, lymphatic metastasis and the presence of ulceration (Figure 3M). Morever, survival analysis showed that the high group had a significantly worse prognosis compared to the low group (Figure 3N). These findings further support that *SPP1* ^+^ macrophage may play an important role in the progression of melanoma.

To explore microenvironmental factors associated with TAM polarisation, we evaluated tumour hallmarks, performed cell‒cell communication analysis and integrated available spatial transcriptomic data. CellChat analysis revealed that skin tumours have some shared macrophage‐associated signalling such as MK signalling (Figure S4A,B). In addition to these common pathways, we also identified cancer‐type‐dominant signalling programs. Notably, SCC tumour cells exhibited the highest interferon response signal among all skin tumour types (Figure S4C). In SCC spatial data, we use mean scores of tumour cell signatures (*KRT5*, *KRT10*, *PTHLH*) to represent tumour cells and *CD68* to represent macrophages. Spatial data showed co‐localisation of *ISG15* (interferon‐responsive genes) in tumour cells with *CXCL9/10* in macrophages, indicating that interferon signalling may drive *CXCL9* ^+^ TAMs polarisation in SCC (Figure S4D,E). Meanwhile, CellChat analysis showed that CM and AM specifically displayed tumour cell‐derived *GDF15* signalling and had stronger signalling strength with *SPP1* ^+^ TAMs than with *CXCL9* ^+^ TAMs (Figure S4A,F). GDF15 has been reported to promote anti‐inflammatory (M2) macrophage polarisation.^39^ For melanoma spatial data, we used mean scores of classical melanoma markers (*PMEL*, *MITF*, *SOX10* and *S100B*) to represent malignant cells (Figure S4G), *CD68* to represent macrophages (Figure S4G,H). Melanoma spatial data showed co‐localisation of *GDF15* in tumour cells and *SPP1* in macrophages, suggesting that *GDF15* might be associated with the *SPP1^+^* macrophage polarisation (Figure S4H).

Collectively, these results delineate that TAMs originate from both monocytes and tissue‐resident macrophages, and they polarise into either tissue‐remodelling or immune‐activating states, depending on the tumour type and stage. Importantly, Mono/Macro subset signatures stratify clinical outcomes and immunotherapy responses, suggesting their potential utility in guiding precision immunotherapy.

### 3.5 T cells display tumour‐ and stage‐specific activation and exhaustion states in skin tumours

**FIGURE 4:**
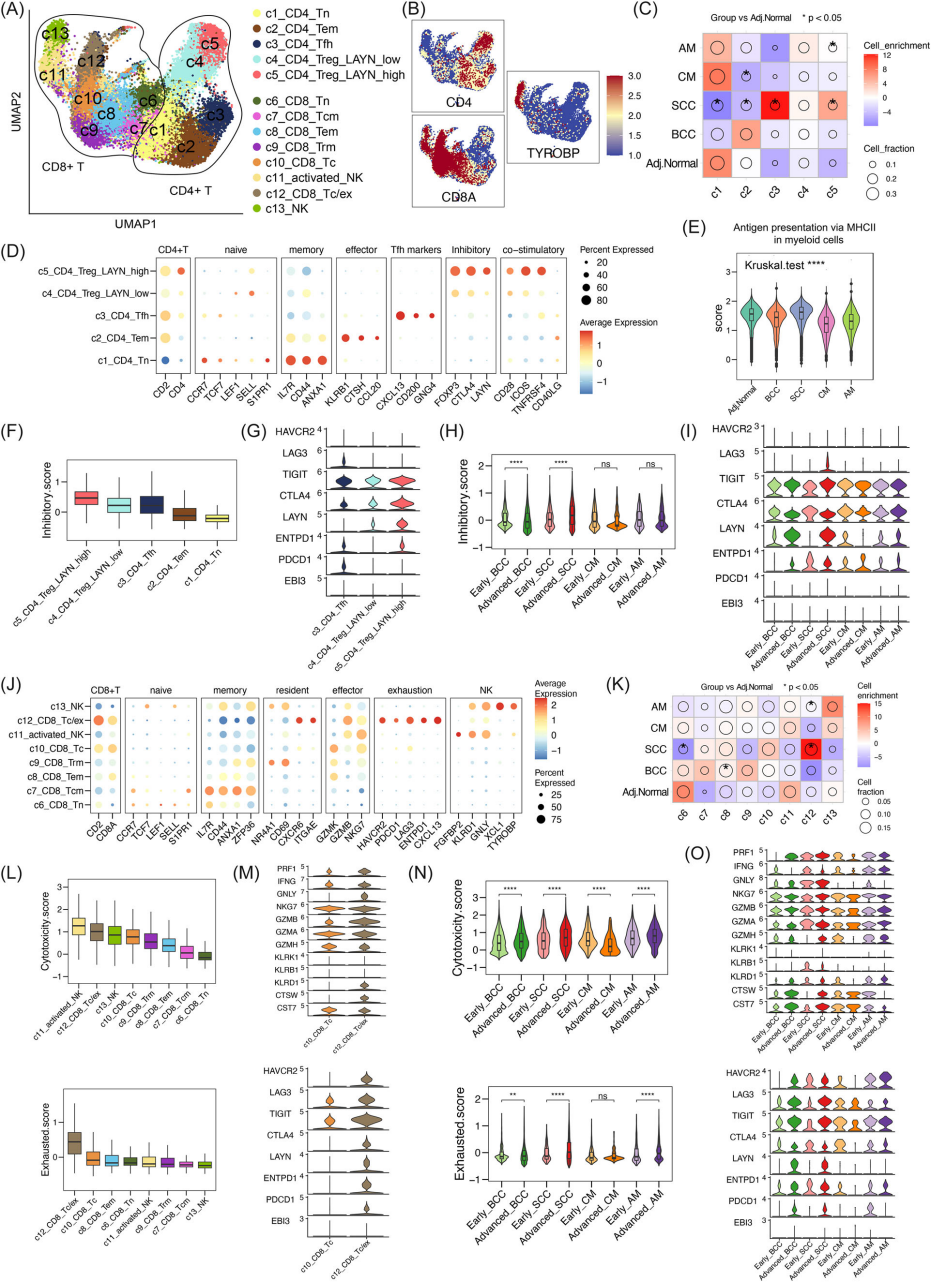
Landscape of T‐ and natural killer (NK)‐cell subsets in skin tumours. (A) Uniform manifold approximation and projection (UMAP) plot of CD4^+^ and CD8^+^T/NK subsets. (B) Feature plots showing the expression of CD4, CD8A and TYROBP. (C) Distribution enrichment analysis of CD4^+^ T‐cell subsets across groups. The size of a circle indicates the relative proportion of cell subsets, colour shows the enrichment level (Pearson residual), and an asterisk denotes a significant change from adjacent normal skin (*p* < .05). (D) Dot plots showing the expression of canonical markers in CD4^+^ T‐cell subsets. (E) Violin plots showing functional scores of myeloid cells across skin tumour groups. (F) Boxplots of inhibitory scores across CD4^+^ subsets. (G) Violin plots of inhibitory checkpoint genes across CD4^+^ T subsets. (H) Boxplots of inhibitory scores in CD4^+^ subsets across tumour groups and stages. (I) Violin plots of inhibitory markers in CD4^+^ subsets across tumour groups and stages. (J) Dot plots showing the expression of canonical markers across CD8^+^T/NK subsets. (K) Distribution enrichment of CD8^+^T/NK subsets across groups. The size of a circle indicates the relative proportion of cell subsets, colour shows the enrichment level (Pearson residual), and an asterisk denotes a significant change from adjacent normal skin (*p* < .05). (L) Boxplots of cytotoxicity and exhaustion scores across CD8^+^T/NK subsets. (M) Violin plots of the expression of cytotoxicity‐associated (top) and exhaustion‐associated (bottom) markers across CD8^+^T/NK subsets. (N) Violin plots of cytotoxicity and exhaustion scores of CD8^+^ subsets across tumour groups and stages. (O) Violin plots of the expression of cytotoxicity‐associated (top) and exhaustion‐associated (bottom) genes of CD8^+^Tc/ex subset across tumour groups and stages. Statistical analyses were performed by two‐sided Wilcoxon rank‐sum test. ^*^ *p* < .05, ^**^ *p* < .01, ^***^ *p* < .001, ^****^ *p* < .0001. Tc/ex, cytotoxic/exhausted T cells; Tcm, central memory T cells; Tem, effect memory T cells; Tfh, follicular helper T cells; Tn, naïve T cells; Treg, regulatory T cells; Trm, tissue‐resident memory T cells.

Our single‐cell transcriptomic analysis revealed 13 distinct T/NK‐cell clusters, including five CD4^+^ T‐cell subsets, six CD8^+^ T‐cell subsets and two NK‐cell subsets (Figure 4A,B). CD4^+^ T‐cell lineages included naïve T cells (c1_CD4_Tn: *CCR7*, *SELL* ^+^), effector memory T cells (c2_CD4_Tem: *IL7R*, *KLRB1* ^+^), follicular helper T cells (c3_CD4_Tfh: *CXCL13*, *CD200* ^+^) and two regulatory T‐cell subsets differing in LAYN expression (c4_CD4_Treg_LAYN_low and c5_CD4_Treg_LAYN_high: *FOXP3*, *LAYN^+^*) (Figure 4A–D). In SCC, we observed a marked shift in CD4^+^ composition when compared with adjacent normal skin. Naïve and effector memory populations decreased, whereas follicular helper T and LAYN^high^ regulatory T‐cell populations increased, indicating a more differentiated CD4^+^ T‐cell phenotypes in SCC (Figure 4C). Consistently, SCC‐derived myeloid cells displayed the highest MHC‐II antigen presentation scores among groups, implicating enhanced antigen presentation as a potential driver of T‐cell differentiation (Figure 4E). In addition, c5_CD4_Treg_LAYN_high was also enriched in AM samples. c5_CD4_Treg_LAYN_high showed the strongest inhibitory scores and highest expression of LAYN, TIGIT and CTLA4 among clusters (Figures 4F,G and S5A), with LAYN expression increasing from early to advanced stages in both BCC and SCC (Figure 4H,I).

Four CD8^+^T/NK lineage subsets were highlighted: cytotoxic T cells (c10_CD8_Tc: *CD8*, *GZMK^+^*), cytotoxic/exhausted T‐cell subset (c12_CD8_Tc/ex: *GZMK*, *LAG3^+^*) and NK cells (c11_activated_NK and c13_NK: *KLRD1*, *GNLY^+^*) (Figure 4J). The proportion of c12_CD8_Tc/ex subset was significantly increased in SCC and AM relative to the adjacent normal skin group (Figure 4K). Functionally, c12_CD8_Tc/ex cells exhibited high cytotoxicity but concomitant exhaustion, whereas NK cells maintained high cytotoxicity with low exhaustion, suggesting relative resistance of NK cells to terminal dysfunction (Figure 4L,M). Across tumour stages, CD8^+^ T cells displayed changes in cytotoxicity and exhaustion states, indicating T‐cell remodelling during tumour progression (Figures 4N and S5B,C). LAYN expression significantly increased in c12_CD8_Tc/ex from early to advanced BCC and SCC, underscoring its association with T‐cell exhaustion (Figure 4O).

Together, these findings reveal the tumour‐type‐ and stage‐specific states of T and NK cells. Notably, c5_CD4_Treg_LAYN_high and c12_CD8_Tc/ex subsets have immunosuppressive roles. Our results also point to LAYN as a potential immunotherapeutic target, particularly in advanced BCC and SCC.

### 3.6 Fibroblasts and endothelial cells are functionally heterogeneous in skin TMEs

**FIGURE 5:**
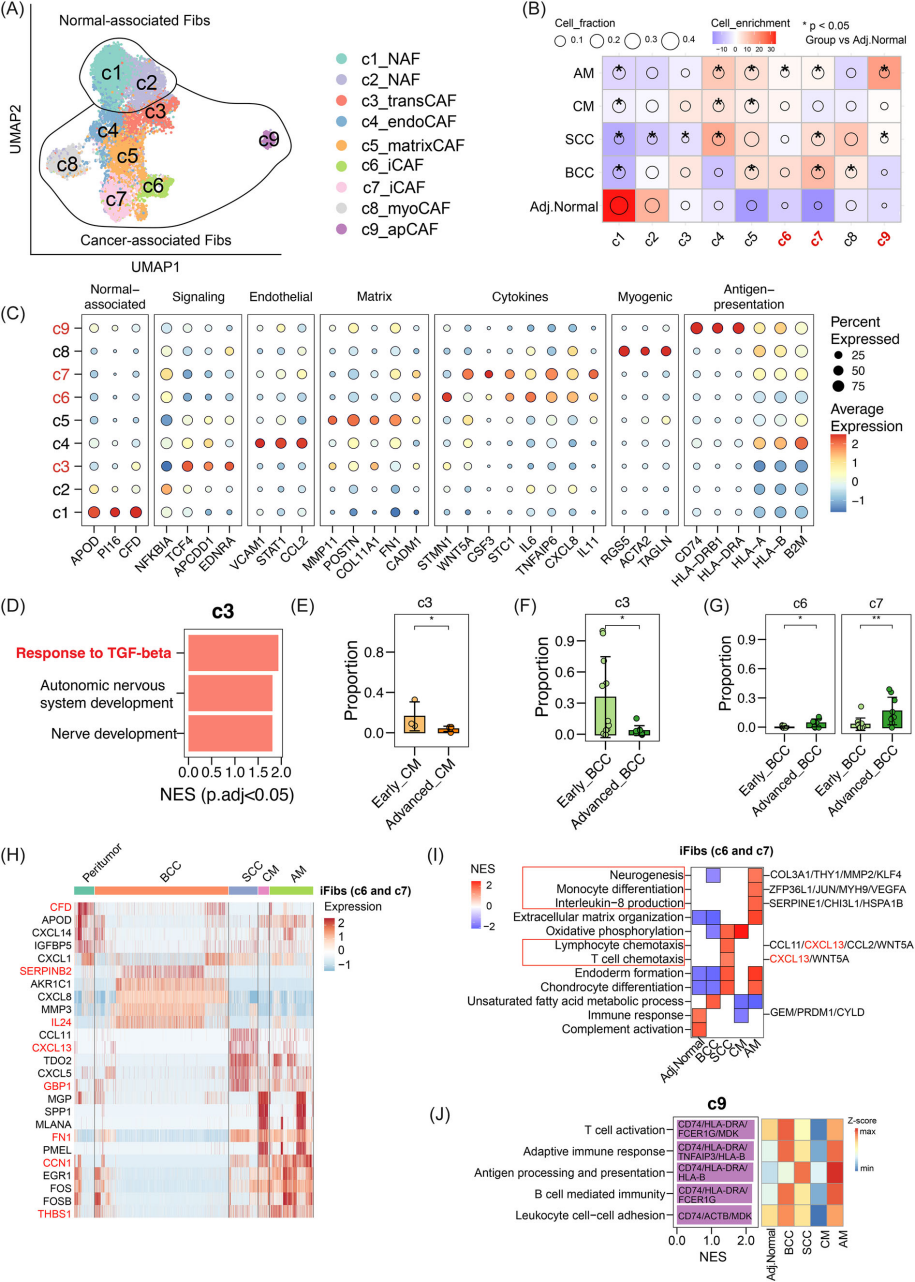
Fibroblast heterogeneity and functional specialisation in skin tumours. (A) Uniform manifold approximation and projection (UMAP) plot of normal‐associated fibroblasts (NAFs) and cancer‐associated fibroblasts (CAFs). (B) Distribution enrichment analysis of fibroblast cell subsets across groups. The size of a circle indicates the relative proportion of cell subsets, colour shows the enrichment level (Pearson residual), and an asterisk denotes a significant change from adjacent normal skin (*p* < .05). (C) Dot plots of differentially expressed genes and associated programs of fibroblast subsets. (D) Functional enrichment analysis of transitional fibroblasts (c3_transFib) compared to other clusters. (E‒G) Bar plots showing proportion comparisons in selected fibroblast subsets. *p*‐Values were calculated using two‐sided Wilcoxon rank‐sum test. Data represent mean ± SD. ^*^ *p* < .05, ^**^ *p* < .01. (H) Heatmap showing differentially expressed genes across fibroblast subsets. (I) Functional enrichment analysis highlighting cytokine signalling pathways of inflammatory cancer‐associated (c6_iCAF and c7_iCAF) across skin tumour groups. (J) Functional enrichment analysis of antigen‐presentation CAFs (c9_apCAF) and functional scores across skin tumour groups. apCAF, antigen‐presentation cancer‐associated fibroblast; endoCAF, endothelial cancer‐associated fibroblast; iCAF, inflammatory cancer‐associated fibroblast; myoFib, myogenic cancer‐associated fibroblast; transCAF, transitional cancer‐associated fibroblast.

Single‐cell transcriptomic analysis revealed multiple fibroblast subsets with distinct tissue preferences and profiles across skin tumours (Figure 5A). Fibroblasts were classified into normal‐associated fibroblasts (NAFs) and CAFs. c1_NAF (*APOD*, *PI16* ^+^) and c2_NAF (*NFKBIA*, *APOD*, *PI16* ^+^) were predominantly enriched in adjacent normal skin group, and CAFs were enriched in skin tumour groups (Figure 5B). CAFs were further stratified into several functionally specialised clusters: transitional (c3_transCAF: *TCF4*, *APCDD1^+^*), endothelial‐like (c4_endoCAF: *VCAM1*, *CCL2^+^*), matrix remodelling (c5_matrixCAF: *MMP11*, *POSTN^+^*), inflammatory (c6_iCAF: *IL6*, *CXCL8* ^+^; c7_iCAF: *CSF3*, *IL6*, *CXCL8* ^+^), myofibroblast‐like (c8_myoCAF: *RGS5*, *ACTA2^+^*) and antigen‐presenting fibroblasts (c9_apCAF: *CD74*, *HLA‐DRB1^+^*) (Figure 5C).

Among these, the c3_transFib show high expression of the stemness gene *TCF4* and signal gene *APCDD1* and exhibited strong enrichment for ‘TGF‐β signalling’ and ‘nerve development’ pathways (Figure 5C,D). The abundance of c3_transFib significantly increased in early‐stage BCC and CM, but decreased in advanced stages, suggesting a potential transitional CAF phenotype (Figure 5E,F).

The inflammatory fibroblast subsets c6_iCAF and c7_iCAF were marked by elevated expression of cytokines and chemokines (Figure 5C), indicating potential immunoregulatory functions. Notably, both subsets were abundant in advanced BCC but less so in early‐stage tumours (Figure 5G), highlighting their role in tumour progression. Further stratification by tumour type revealed divergent cytokine expression patterns across iCAFs: *CFD* and *CXCL14* in adjacent normal skin tissue, *SERPINB2* and *IL24* in BCC group, *CXCL13* and *GBP1* in SCC group, *FN1* in CM group, and *EGR1* and *THBS1* in AM group (Figure 5H). Functional enrichment analysis indicated that SCC‐associated iCAFs were involved in ‘T‐cell chemotaxis’, while AM‐associated iCAFs were linked to ‘neurogenesis’ and ‘monocyte differentiation’, underscoring tumour‐type‐specific immunomodulatory roles (Figure 5I). Additionally, c9_apCAF, characterised by antigen presentation‐associated genes, was notably enriched in AM, suggesting a unique role in immune regulation in this tumour subtype (Figure 5B,J).

Endothelial cells within skin tumours were classified into seven distinct clusters, comprising one artery, four capillary, one venous and one lymphatic endothelial subtypes (Figure S6A‒C). The c2_capillary subset was significantly enriched across all tumour types relative to the adjacent normal skin group and expressed high levels of *COL4A1*, *COL4A2* and *RGCC* (Figure S6B,D). The c2_capillary subset was enriched in ‘extracellular matrix organisation’ and ‘angiogenesis’ pathways (Figure S6E). Lymphatic endothelial cells exhibited tumour‐specific transcriptional features, with enrichment of CD34 and KLF2 in AM, along with ‘activation of myeloid differentiation’ and ‘ERK signalling pathways’, suggesting context‐dependent functional adaptations (Figure S6F,G).

Overall, these results reveal a complex heterogeneity of stromal components, including fibroblasts and endothelial cells, across various skin tumour types, suggesting their functional programming in immune regulation and stromal remodelling.

### 3.7 Ecotypes reflect stage‐associated progression patterns in skin tumours

**FIGURE 6:**
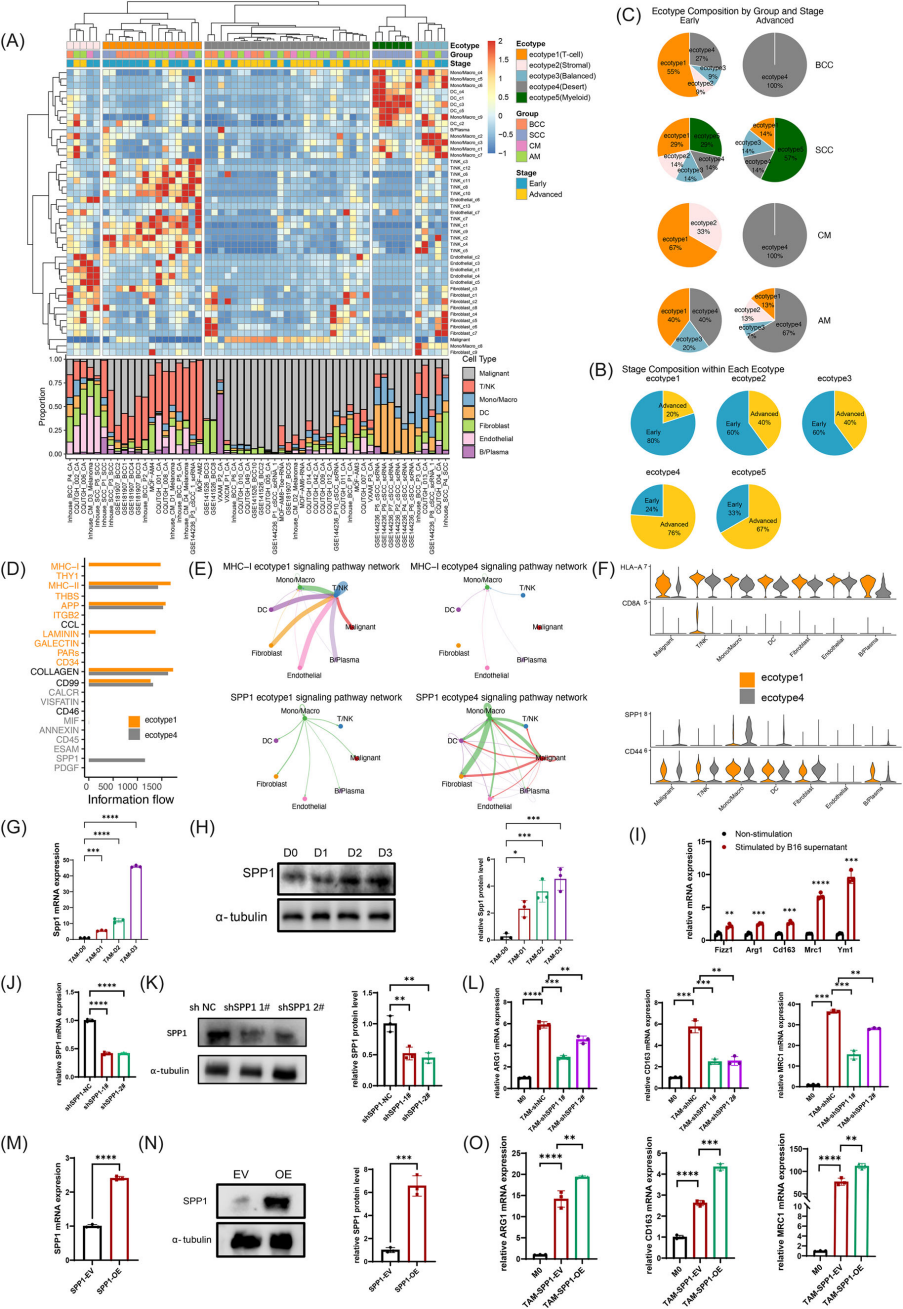
Ecotypes and cell‒cell communication networks in skin tumour microenvironment. (A) Heatmap showing the five ecotypes of skin tumours, which were inferred based on tumour microenvironment cell compositions. Bar plot showing the distribution of various cell lineages in each sample. (B) Pie charts showing ecotype distribution across tumour types and stages. (C) Pie charts showing tumour‐stage compositions across ecotypes. (D) Bar plots of enriched ligand‒receptor interactions for ecotype 1 and ecotype 4. (E) Cell‒cell communication networks showing MHC‐I signalling interactions in ecotype 1 and ecotype 4 (top) and *SPP1* signalling interactions in ecotype 1 and ecotype 4 (bottom). Edge thickness indicates interaction strength, and colours represent different cell lineages. (F) Violin plots showing the expression of HLA‐A (top) and SPP1 (bottom) with associated partner genes (CD8A, CD44) across cell types and ecotypes. (G) qRT‐PCR showing *Spp1* expression in RAW264.7 macrophages cultured with conditioned medium from B16 melanoma cells. (H) Western blot showing Spp1 protein levels in tumour‐associated macrophages (TAMs) after B16‐conditioned medium treatment, with corresponding quantification. (I) qRT‐PCR showing M2 polarisation markers expression in RAW264.7 macrophages after B16‐conditioned medium treatment. (J) qRT‐PCR showing SPP1 mRNA expression in THP‐1 macrophages transduced with control short hairpin RNA (shRNA) (negative control shRNA [shNC]) or two independent *SPP1*‐targeting shRNAs (shSPP1‐1# and shSPP1‐2#) (K) Western blot showing *SPP1* protein levels in shNC, sh*SPP1*‐1# and sh*SPP1*‐2# THP‐1 cells. (L) M2 polarisation markers (*ARG‐1*, *MRC1* and *CD163)* were measured in shNC or sh*SPP1* THP‐1 macrophages cultured with SK‐MEL‐28‐conditioned medium. (M) qRT‐PCR showing *SPP1* mRNA expression in THP‐1 macrophages transduced with empty vector (EV) or *SPP1* overexpression construct (SPP1‐OE). (N) Western blot showing *SPP1* protein levels in EV and *SPP1*‐OE THP‐1 cells. (O) M2 polarisation markers (*ARG‐1*, *MRC1* and *CD163*) were measured in EV/*SPP1*‐OE THP‐1 macrophages cultured with SK‐MEL‐28‐conditioned medium. Data are presented as mean ± SD. *n* = 3 independent repeats. Unpaired, two‐tailed *t*‐test; ^*^ *p* < .05, ^**^ *p* < .01, ^***^ *p* < .001, ^****^ *p* < .0001.

To further understand the tumour immune landscape, we investigated the cellular ecotypes within the skin tumour ecosystem, which encompassed 44 cell subtypes. Utilising unsupervised hierarchical clustering analysis based on relative cellular abundance, we identified five ecotypes representing distinct cellular compositions and co‐association patterns (Figure 6A–C): ecotype 1: T‐cell‐dominant type, with abundant T lymphocytes; ecotype 2: stromal‐enriched type, with a higher proportion of fibroblasts and endothelial cells; ecotype 3: balanced type, with relatively balanced components; ecotype 4: desert type, dominated by tumour cells; ecotype 5: myeloid‐enriched type, prominently composed of infiltrated myeloid cells.

We then investigated the association between ecotypes and clinical parameters, including tumour types and stages. T‐cell‐dominant ecotypes (E1) were mainly composed of early‐stage tumours (80%), while desert ecotypes (E4) were mainly composed of advanced‐stage tumours (76%) (Figure 6B). Analysis across tumour types and stages revealed distinct distribution patterns. The desert ecotype (E4) was the major component of the advanced‐stage BCC, CM and AM, while myeloid‐enriched ecotype (E5) was the prominent ecotype in advanced SCC, thus revealing that SCC significantly differs from other skin tumours. Moreover, the myeloid‐enriched ecotype was 29% in the early‐stage SCC and increased to 57% in the advanced stage, suggesting that this ecotype plays a crucial role in the progression of SCC tumours. Therefore, patients with SCC may particularly benefit from myeloid‐targeted immunotherapeutic strategies. In BCC, early‐stage samples were dominated by the T‐cell‐dominant type (E1, 67%), but shifted entirely to the desert ecotypes (E4, 100%) in advanced stages. In CM, early‐stage samples were composed of the T‐cell‐dominant type (E1, 67%) and the stromal type (E2, 33%); however, all advanced‐stage CM samples shifted to the desert type (E4, 100%). In AM, both early‐ and advanced‐stage tumours showed high proportions of the desert type (E4, 40% and 67%, respectively) (Figure 6C). These observations suggest that ecotype‐based classification reveals clinically meaningful differences in the immune microenvironment, offering novel insights into oncogenesis and informing the choice of immunotherapy for skin cancers.

To validate the spatial organisation of immune ecotypes, we inquired about spatial transcriptomic data for SCC and melanoma samples. We examined representative gene expression, including mean scores of tumour cell signatures, as well as immune markers such as *PTPRC* for immune cells, *CD3D* for T cells, *CD68* for macrophages and *LAMP3* for DCs. Two SCC samples in the scRNA‐seq cohort were accompanied by matched spatial transcriptomic data and were both classified into ‘myeloid’ ecotype. In the representative SCC sample, myeloid markers (*CD68* and *LAMP3*) were detected in both tumour core and tumour margin regions, indicating extensive myeloid infiltration throughout the tumour tissue (Figure S7A,B). In the melanoma sample,^40^ immune‐related transcripts were predominantly localised to tumour margins, consistent with an immune‐excluded configuration (Figure S7B). Together, these spatial analyses provide validation of the representative immune ecotypes, while larger cohorts are needed to comprehensively illustrate the spatial atlas of skin tumours in the future.

### 3.8 *SPP1* signalling in macrophages contributes to the desert ecotype of TME and regulates M2 polarisation

Given that the T‐cell‐dominant ecotype predominates in early‐stage tumours, whereas the desert ecotype typifies advanced‐stage tumours, we next performed cell‒cell communication analyses to compare these two ecotypes. The results revealed that the T‐cell‐dominant ecotype was prominently enriched for MHC‐I signalling pathways, whereas the desert ecotype was predominantly governed by *SPP1* signalling (Figure 6D,E). Specifically, malignant cells in the T‐cell‐dominant ecotype exhibited higher expression of MHC‐I molecules (e.g., HLA‐A), accompanied by markedly elevated CD8A expression in T/NK cells, suggesting enhanced antigen presentation and cytotoxic T‐cell activation. In contrast, Mono/Macro populations showed increased SPP1 expression in the desert ecotype compared to the T‐cell‐dominant ecotype, suggesting the important role of *SPP1* ^+^ macrophages in TME remodelling (Figure 6F).

**FIGURE 7:**
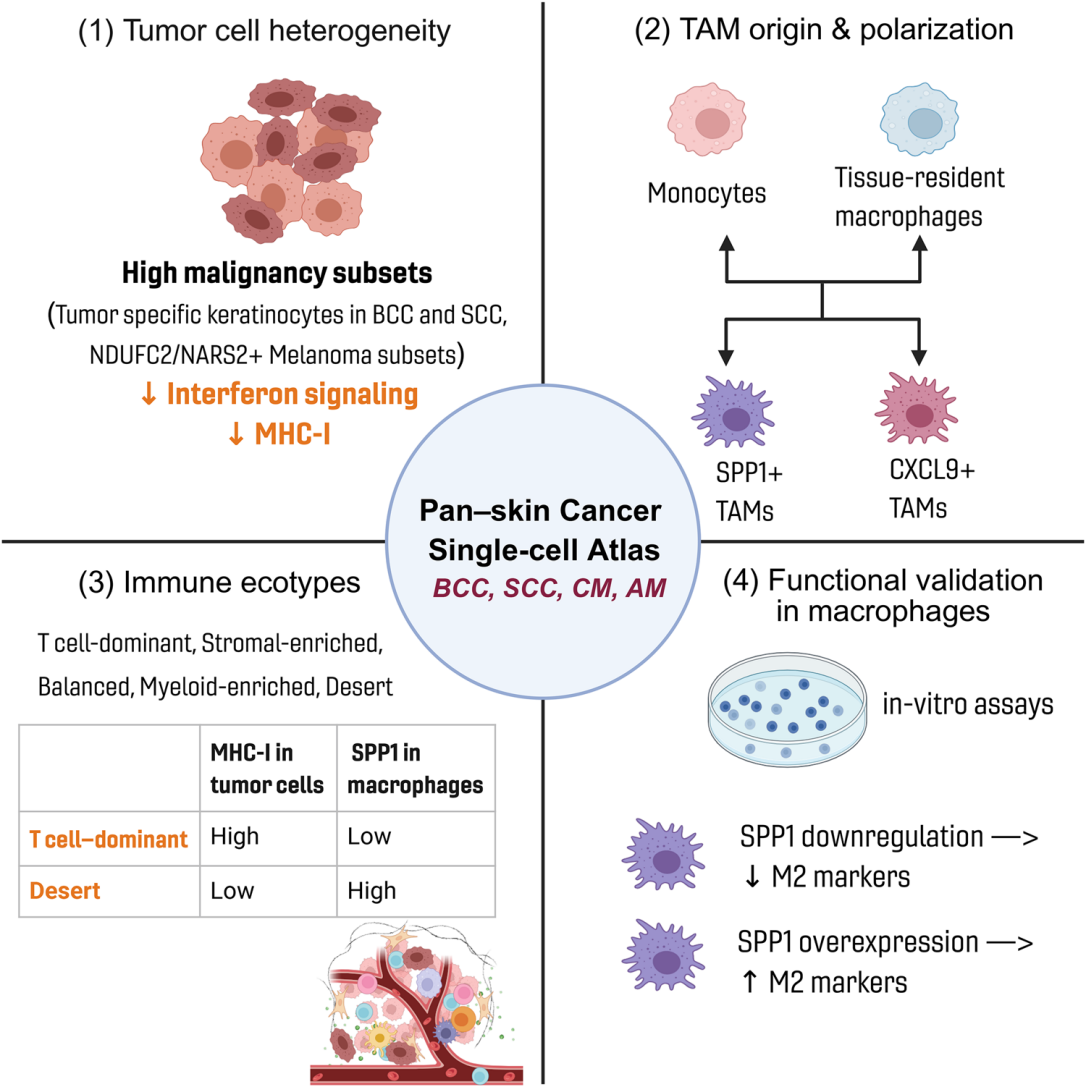
Integrative single‐cell analysis of pan‐skin cancers. The figure points out the key findings of this research: (1) distinct tumour cell subpopulations, in which malignancy‐associated clusters exhibit reduced interferon signalling and MHC class I expression. (2) Tumour‐associated macrophages (TAMs) derived from both monocyte‐derived and tissue‐resident lineages segregate into two mutually exclusive polarisation states, SPP1^+^ and CXCL9^+^. (3) Five immune ecotypes are defined. Comparisons between T‐cell‐dominant and desert ecotypes shows that T‐cell‐dominant ecotype have higher MHC‐I expression in tumour cells, whereas desert ecotypes have higher SPP1^+^ TAMs. (4) In vitro assays showed that SPP1 knockdown reduces M2 markers while SPP1 overexpression enhances them in macrophages. In the figure, ‘↑’ indicates upregulation and ‘↓’ indicates downregulation.

Next, we employed melanoma models to explore the relationships among *SPP1*, tumour cells and macrophage polarisation in TME. RAW264.7 macrophages were cultured in conditioned medium derived from the B16‐F10 melanoma cell line, resulting in a progressive upregulation of *Spp1* expression with prolonged exposure (Figure 6G,H). Concomitantly, B16‐F10‐conditioned medium induced increased expression of M2 polarisation markers, including *Mrc1*, *Cd163* and *Ym1*, in RAW264.7 macrophages. These findings were further validated using conditioned medium from the Yumm1.7 melanoma cell line (Figure S7E,F). To investigate the role of *SPP1* in macrophage polarisation, we conducted *SPP1* knockdown and overexpression experiments in the human monocytic cell line THP‐1. Quantitative PCR and Western blot confirmed efficient suppression (Figure 6J,K) or overexpression (Figure 6M,N) of *SPP1* at both mRNA and protein levels. These THP‐1 cells were then differentiated into macrophages by PMA stimulation and further differentiated into TAMs using conditioned medium from the human melanoma cell line SK‐MEL‐28. Notably, knockdown of *SPP1* in THP‐1‐derived TAMs led to downregulation of M2 polarisation markers (*ARG1*, *CD163* and *MRC1*), whereas overexpression of *SPP1* led to upregulated expression of M2 polarisation markers (Figure 6L,O). Together, these findings demonstrate that *SPP1* could regulate macrophage M2 polarisation, contributing to the TME remodelling during tumour progression(Figure 7).

## 4 DISCUSSION

In this study, we present an integrative single‐cell transcriptomic atlas of BCC, SCC, CM and AM, spanning both early‐ and advanced‐stage skin tumours. Multi‐omics analyses highlight both shared and specific immune remodelling programs across skin cancers, characterised by the emergence of immune‐evasive tumour subsets, divergent macrophage polarisation states and stage‐linked transitions in immune ecotypes.

Increasing evidence indicates that intratumoural heterogeneity is closely linked to prognosis such as survival and immunotherapy responses.^41^ One of our notable findings is the identification of a *NARS2^+^NDUFC2* ^+^ melanoma cell subpopulation, characterised by reduced MHC‐I expression and poor prognosis. Furthermore, CRISPR‐based dependency screens indicate that NARS2 and NDUFC2 are necessary for tumour cell proliferation. *NDUFC2* and *NARS2* are key regulators of mitochondrial respiratory function and protein synthesis. Notably, the *NARS2/NDUFC2* ^+^ c1_Mel subpopulation exhibits high mitochondrial activity and energy‐metabolic state. Previous studies have demonstrated that mitochondrial function, including complex I activity and metabolic status, can significantly influence tumour immunogenicity and MHC‐I expression.^42^, ^43^, ^44^ These findings suggest a potential mechanism that *NARS2/NDUFC2*‐associated mitochondrial metabolic programs may contribute to altered MHC‐I expression in melanoma.^45^, ^46^ We believe that future studies dissecting how mitochondrial metabolism shapes antigen presentation machinery will be of high interest and may provide novel therapeutic opportunities to restore tumour immunogenicity.

Our dissection of the myeloid compartment revealed that TAMs arise from both circulating monocytes and tissue‐resident macrophages, polarising towards either immunosuppressive tissue‐remodelling *SPP1* ^+^ states or pro‐inflammatory *CXCL9^+^CXCL10* ^+^ states. While previous studies have described macrophage heterogeneity in skin cancers,^7^, ^9^ our work adds a lineage‐tracing perspective, demonstrating distinct ontogenies that shape their functional polarisation. Notably, the functional antagonism between *SPP1* ^+^ and *CXCL9^+^/CXCL10* ^+^ TAMs provide a mechanistic explanation for the dynamic balance of immunosuppressive versus immune‐activating programs across different skin cancer types, and further explains the differences in malignancy. Similar trends have been reported previously. Bill et al. identified CXCL9:*SPP1* polarity as a key predictor of prognosis in pan‐cancer.^47^ In our in‐house IF staining cohort, *SPP1* ^+^ TAM infiltration correlates with worse clinical features such as clinical stage, metastasis and poor patient survival. Meanwhile, *SPP1* ^+^ macrophages were identified as crucial component difference between ‘T‐cell‐dominant’ ecotype and ‘desert’ ecotype and could regulate M2 macrophage polarisation. Overall, these results underscore the potential of *SPP1* ^+^ macrophages as actionable targets of cancer therapy.

We further propose an ecotype‐based classification of skin cancer immune microenvironments, comprising T‐cell‐dominant, stromal‐enriched, balanced, desert and myeloid‐enriched ecotypes. This framework refines the conventional dichotomy of ‘hot’ versus ‘cold’ tumours.^48^ While BCC, CM and AM predominantly transition from T‐cell‐dominant to immune‐desert ecotypes during progression, SCC uniquely shifts towards a myeloid‐enriched state. CellChat analysis revealed enrichment of IL1 signalling in SCC, which has been reported to recruit monocyte and macrophage into tissues.^36^ Together with signalling such as *MDK*, *SPP1* and interferon, these signals form a complex macrophage regulation network in SCC, providing potential therapeutic targets such as myeloid‐target therapy for SCC.

While our ecotype analysis highlights the immune heterogeneity across skin cancers, increasing evidence suggests that TME states are shaped by additional regulatory layers, including metabolic and microbial influences.^49^, ^50^ From a therapeutic perspective, growing attention has shifted towards strategies that actively remodel the TME. Nanomaterial‐based platforms and radiopharmaceutical approaches could precisely modulate immune and stromal components in TME and may help overcome immune exclusion or myeloid‐dominated suppression in our analysis.^51^, ^52^ Emerging work showed that NK‐cell‐based strategies is particularly suitable for tumour subsets exhibiting impaired antigen presentation and immune escape.^53^ Notably, DCs constituted a substantial fraction of immune cells in our dataset. DCs encompass multiple functionally distinct subsets such as conventional DCs and LAMP3+ DCs, and the skin additionally harbours tissue‐resident DCs (Langerhans cells).^54^ Future directions could focus on the heterogeneity of DCs, their associations with clinical outcomes, and potential therapeutic targets.

Despite these remarkable new insights, several limitations exist in our studies. First, the sample size of this cohort, although the largest among the profiled to date, remains modest, and further validation in larger cohorts is necessary to generalise the observed ecotype patterns. Second, while our in vitro models confirmed melanoma‒macrophage interactions, in vivo lineage‐tracing studies are required to definitively resolve TAM ontogeny and to further compare their function polarisation trajectory. Finally, functional testing of therapeutic strategies targeting *SPP1* ^+^ TAMs pathways is crucial to validating their translational potential.

## 5 CONCLUSIONS

Our integrative analysis reveals an atlas of immune remodelling across different skin cancers, identifying both shared and cancer‐type‐specific mechanisms of immune evasion. Importantly, the negative correlation between MHC‐I genes and tumour signatures (e.g., *NARS2*) may enable us to modulate the antigen presentation ability in tumour cells. Diverse phenotypes of macrophages, particularly *SPP1^+^* macrophages, provide potential therapeutic targets for reshaping the immune status of the TME. These findings underscore the need to tailor therapeutic strategies to the distinct immune ecologies of skin cancers, providing a foundation for biomarker discovery and the development of new intervention strategies.

## AUTHOR CONTRIBUTIONS

Lingjuan Huang performed the data analysis and prepared the figures. Huihui Hou collected and curated the data and carried out validation experiments. Xiyuan Zhang, Shiyao Pei and Liang Dong conducted experiments to produce the data. Jie Sun, Wensheng Shi and Xin Li processed sequencing data. Anil Prakash, Mason Liu, Haoqiu Song and Shenglin Mei provided supervision and data interpretation. Xiang Chen and Mingzhu Yin acquired funding and provided resources. Lingjuan Huang and Huihui Hou drafted the manuscript. Shenglin Mei, Mingzhu Yin and Mason Liu contributed to manuscript revision. Mingzhu Yin and Xiang Chen conceived and designed the study. All the authors read and approved the final version.

## CONFLICT OF INTEREST STATEMENT

The authors declare no conflicts of interest.

## ETHICS STATEMENT

This study was approved by the Ethics Committee of Central South University Xiangya Hospital (approval number: 202202043), and written informed consent was obtained from all the participants. Public data were collected from databases where ethical approval and informed consent had been obtained by the original studies.

## Supporting information



Supporting Information

Supporting Information

Supporting Information

Supporting Information

Supporting Information

Supporting Information

Supporting Information

Supporting Information

Supporting Information

## Data Availability

The scRNA‐seq data presented in this study have been deposited to the Genome Sequence Archive in the National Genomics Data Center (https://ngdc.cncb.ac.cn/?lang=en) under BioProject IDs: PRJCA012942 (BCC), PRJCA025225 (SCC), PRJCA030492 (CM) and PRJCA018695 (CM and AM). Publicly available scRNA‐seq datasets were obtained from Gene Expression Omnibus (GEO, https://www.ncbi.nlm.nih.gov/geo/) under accession codes: GSE141526 (BCC), GSE123813 (BCC and SCC), GSE181907 (BCC), GSE144236 (SCC) and GSE189889 (AM). The bulk RNA‐seq data and clinical information for melanoma samples can be found in the GEO database under accession numbers: GSE19234 and GSE91061. The spatial data were inquired from SpatialTME (https://www.spatialtme.yelab.site/#!/), with sample ID: GSM4565823_P4_rep1 (SCC) and GSM5420750 (melanoma). The analysis code supporting this study is available upon reasonable request.
