# Supplementary material for: Integrative single‐cell analysis uncovers distinct tumour microenvironment ecotypes and immune evasion across skin cancers
Source: Clin Transl Med. 2026 Feb 4;16(2):e70611. doi: 10.1002/ctm2.70611 (PMC12869349; doi:10.1002/ctm2.70611)
Supplement: Supplementary file 1 — Supporting Information [file CTM2-16-e70611-s003.docx]

**Supplementary Materials**

**Figure S1. Integration of skin tumor scRNA-seq datasets.**

Figure S2. Landscape of different tumor cells among different skin tumors

Figure S3. Characterization of myeloid cell subsets in skin tumors.

Figure S4. Myeloid-associated signaling programs and spatial validation across skin cancers.
**Figure S5. Functional scores of T and NK cell subsets in skin tumors.**

**Figure S6. Endothelial cell subsets in skin tumors.**

**Figure S7. Spatial validation of ecotypes and cell-cell communications in skin tumors**

Table S1. Collection of single-cell RNA sequencing datasets.

Table S2. Sample information of single-cell RNA sequencing datasets.

Table S3. Differential expressed genes among melanoma and adjacent normal skin melanocytes subsets.

Table S4. Clinicopathological information of patients in in-house melanoma cohort used for immunofluorescent staining.

Table S5. Sequences of primers used in this study, Related to Methods.

**Figure S1. Integration of skin tumor scRNA-seq datasets.** (A) UMAP plots of individual datasets showing major cell lineages. (B) Integrated UMAP plots colored by sequencing platform before and after batch correction.

**Figure S2. Landscape of different tumor cells among different skin tumors.** (A) Representative inferCNV plots of malignant cells across skin tumor types. (B) Gene set enrichment analysis among different melanoma subpopulations. (C-E) Survival analysis of representative melanoma subsets using GSE19234 (n=44), including c1_Mel (C), c2_Mel (D), and c5_Mel (E). (F) Gene effect scores from DepMap CRISPR screens showing essential genes in cutaneous and acral melanoma cell lines. Gene effect scores were retrieved for each target gene, where a lower score (typically < -0.5) indicates a higher dependency of the cell line on the gene for survival in DepMap. (G) Hallmark pathway activity scores across tumor types and stages. Mel, melanoma and melanocyte subsets.

**Figure S3.** **Characterization of myeloid cell subsets in skin tumors.** (A) UMAP visualization of myeloid cells colored by cell type and technology. (B) Violin plots showing canonical marker gene expression across monocytes, macrophages, and dendritic cells. (C) Functional module scores projected onto UMAP. (D) Violin plots of differentially expressed genes of c2_INHBA_Mono across groups. (E) Functional enrichment of c2_INHBA⁺ monocytes across groups. Heatmap showing normalized enrichment scores (NES) of biological processes across groups. Red indicates positive enrichment; blue indicates negative enrichment. (F) Stemness scores (top) and differentiation potency scores (bottom) of monocyte and macrophage subsets. (G) Proportions of monocyte and macrophage subsets in early versus advanced basal cell carcinoma. Each dot represents a sample. *P* values were calculated using the two-sided Wilcoxon rank-sum test. * *p* < 0.05, ** *p* < 0.01. (H) Heatmap of differentially expressed genes across monocyte/macrophage subsets.

**Figure S4.** **Myeloid-associated signaling programs and spatial validation across skin cancers.** (A) Relative information flow of major ligand–receptor signaling pathways across BCC, SCC, CM, and AM inferred by CellChat. (B) Cell–cell communication networks of the MK (MDK) signaling pathway. Edge thickness indicates signaling strength. (C) Interferon signature scores of adjacent normal and malignant cells across skin cancers. (D) Representative HE slides and spatial transcriptomics section showing tumor marker expression of SCC (KRT5, KRT10, PTHLH). (E) Spatial expression of macrophage markers (CD68) and polarization markers (SPP1 and CXCL9). (F) Cell–cell communication networks of the GDF (GDF15) signaling pathway in CM and AM. (G) Representative HE slides and spatial transcriptomics tumor section showing tumor signature (PMEL, MITF, SOX10, and S100B) distribution in melanoma. (H) Spatial expression of melanoma-markers (S100B), tumor-derived factors (GDF15), macrophage markers (CD68), and macrophage-polarization markers (SPP1 and CXCL9).

**Figure S5. Functional scores of T and NK cell subsets in skin tumors.** (A) Violin plots of inhibitory scores in CD4⁺ T-cell subsets across tumor types and stages. (B) Violin plots of cytotoxicity scores in CD8⁺ T/NK subsets across tumor types and stages. (C) Violin plots of exhaustion scores in CD8⁺ T/NK subsets across tumor types and stages. *P* values were calculated using the two-sided Wilcoxon rank-sum test. * *p* < 0.05, ** *p* < 0.01, *** *p* < 0.001, **** *p* < 0.0001. Tn, naïve T cells; Tem, effect memory T cells; Tfh, follicular helper T cells; T reg, regulatory T cells; Tcm, central memory T cells; Trm, resident memory T cells; Tc/ex, cytotoxic/exhausted T cells; NK, natural killer cells.

**Figure S6. Endothelial cell subsets in skin tumors.** (A) UMAP plot of endothelial cell subsets. (B) Enrichment of vascular endothelial subsets across groups. Circle size indicates relative proportion, color denotes enrichment level, and asterisks mark significant changes versus adjacent normal skin (*p* < 0.05, two-sided Wilcoxon rank-sum test). (C) Feature plots of classical marker genes. (D) Heatmap of differentially expressed genes in vascular endothelial subsets. (E) Heatmap of differentially expressed genes in lymphatic endothelial cells across tumor groups. (F) GO enrichment of vascular endothelial subsets. (G) GO enrichment of lymphatic endothelial cells across tumor groups.

Figure S7. Spatial transcriptomic validation of immune and stromal cell distribution. (A) Representative histological image (upper) and spatial distribution of the tumor signature score in SCC (lower). (B) Spatial expression of immune and stromal cell markers in SCC, including PTPRC for immune cells, CD3T for T cells, CD68 for macrophages, LAMP3 for DCs, LUM for fibroblasts, and PECAM1 for endothelial cells. (C) Representative histological image and spatial distribution of the tumor signature score in melanoma. (D) Spatial expression of immune and stromal cell markers in melanoma, including PTPRC for immune cells, CD3T for T cells, CD68 for macrophages, LAMP3 for DCs, LUM for fibroblasts, and PECAM1 for endothelial cells. (E) qRT-PCR showing M2 polarization markers expression in RAW264.7 macrophages after yumm1.7-conditioned medium treatment. (F) qRT-PCR showing Spp1 expression in RAW264.7 macrophage cell line cultured with conditioned medium from yumm1.7 melanoma cell line (left). Western blot showing Spp1 protein levels in TAMs after yumm1.7-conditioned medium treatment (middle), with corresponding quantification (right).
