# Supplementary figures and images for: Integrative single‐cell analysis uncovers distinct tumour microenvironment ecotypes and immune evasion across skin cancers
Source: Clin Transl Med. 2026 Feb 4;16(2):e70611. doi: 10.1002/ctm2.70611 (PMC12869349; doi:10.1002/ctm2.70611)

Figure S1

A

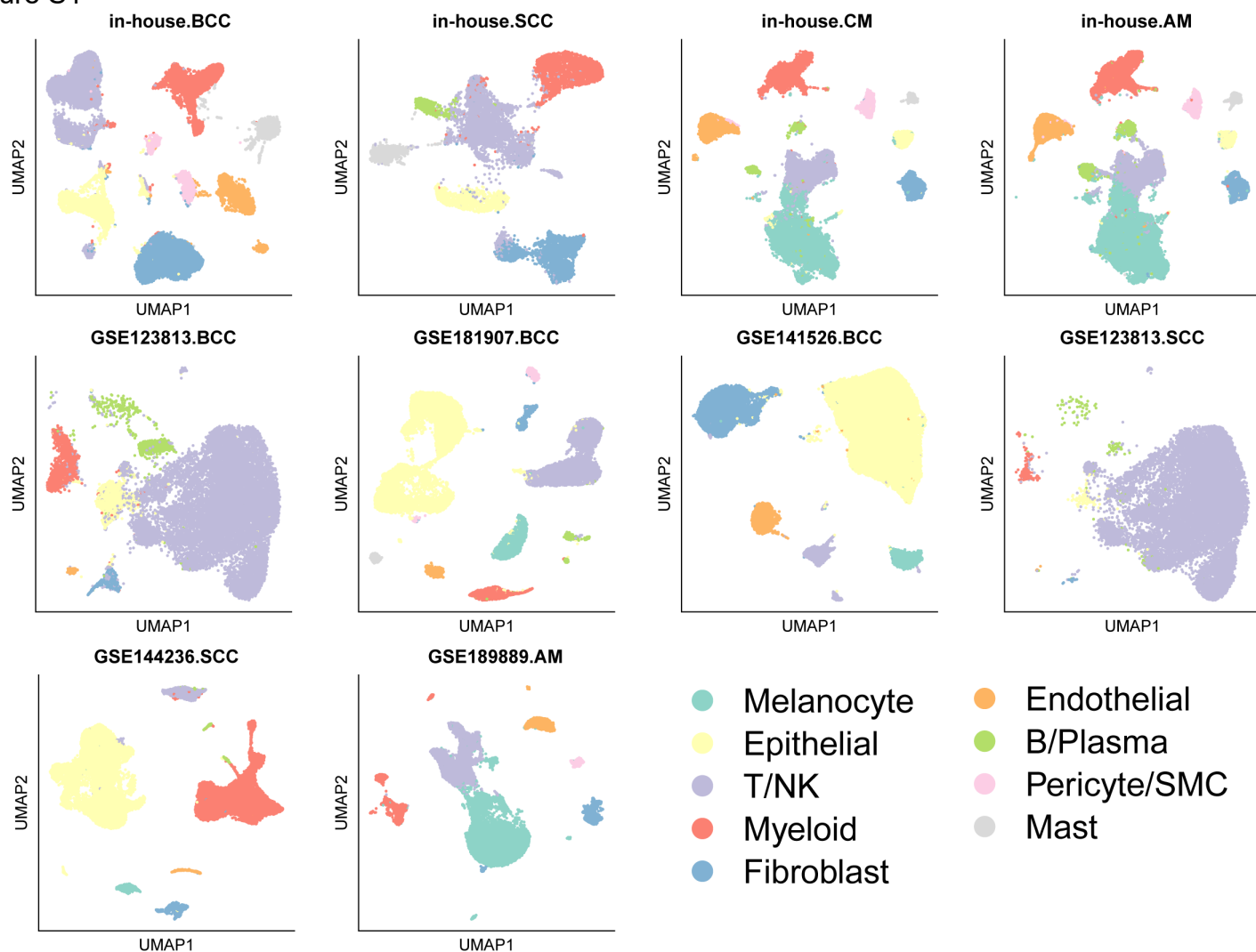

B

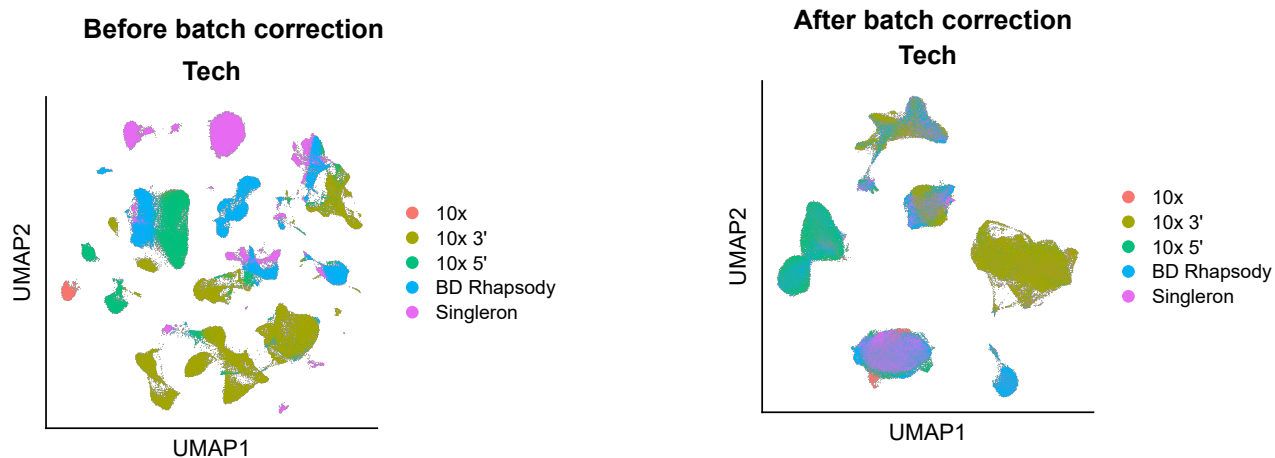

Supplement: Supplementary file 3 — Supporting Information [file CTM2-16-e70611-s001.pdf]

**A** Figure S2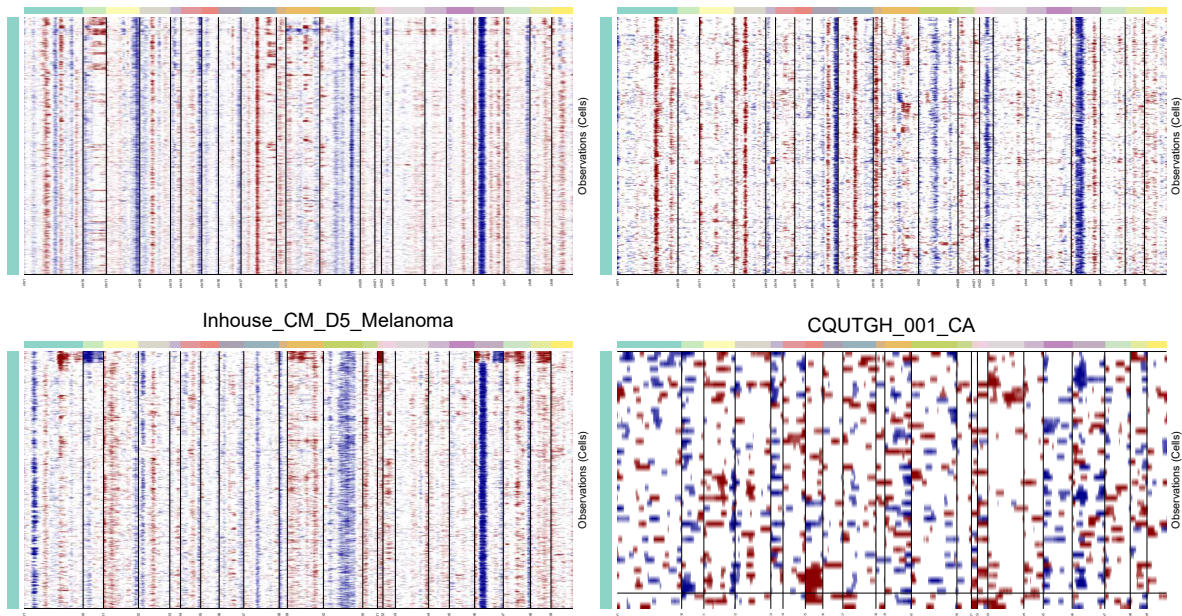**B**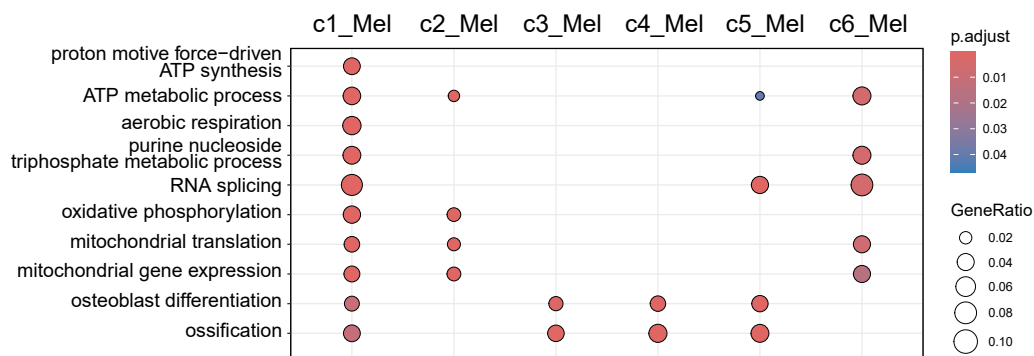**C**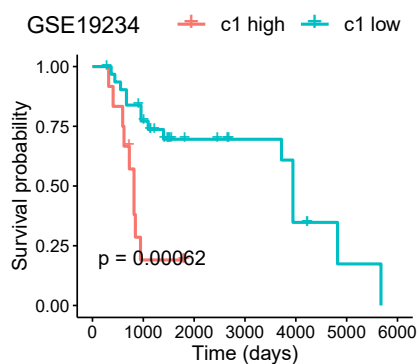**D**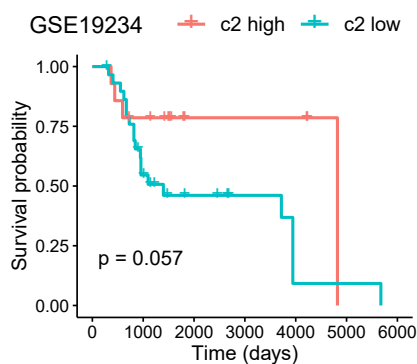**E**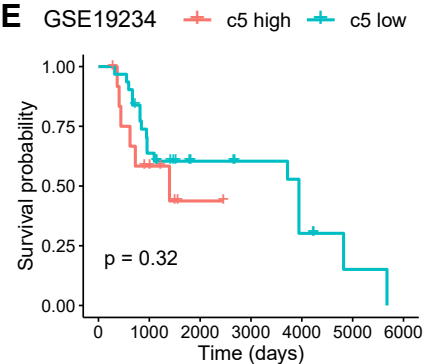**F**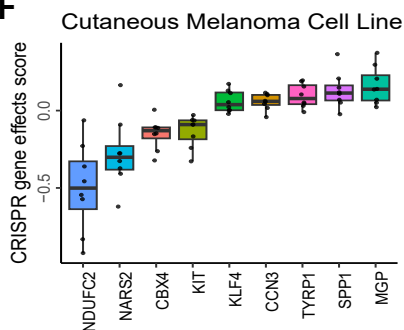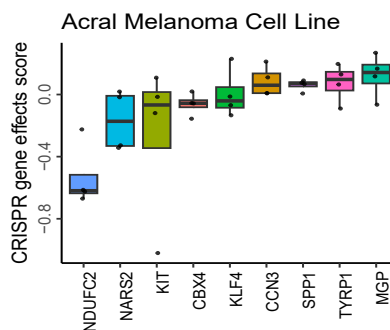**G**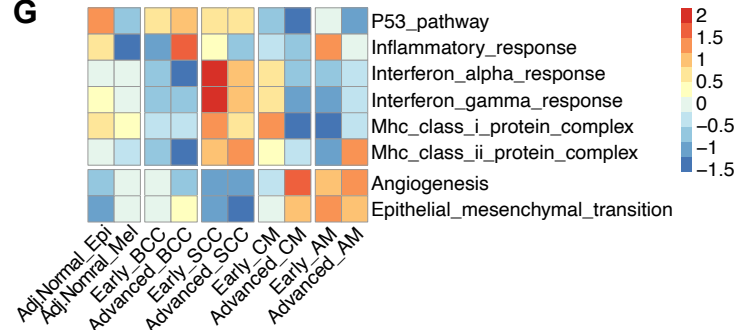

Supplement: Supplementary file 4 — Supporting Information [file CTM2-16-e70611-s002.pdf]

Figure S3

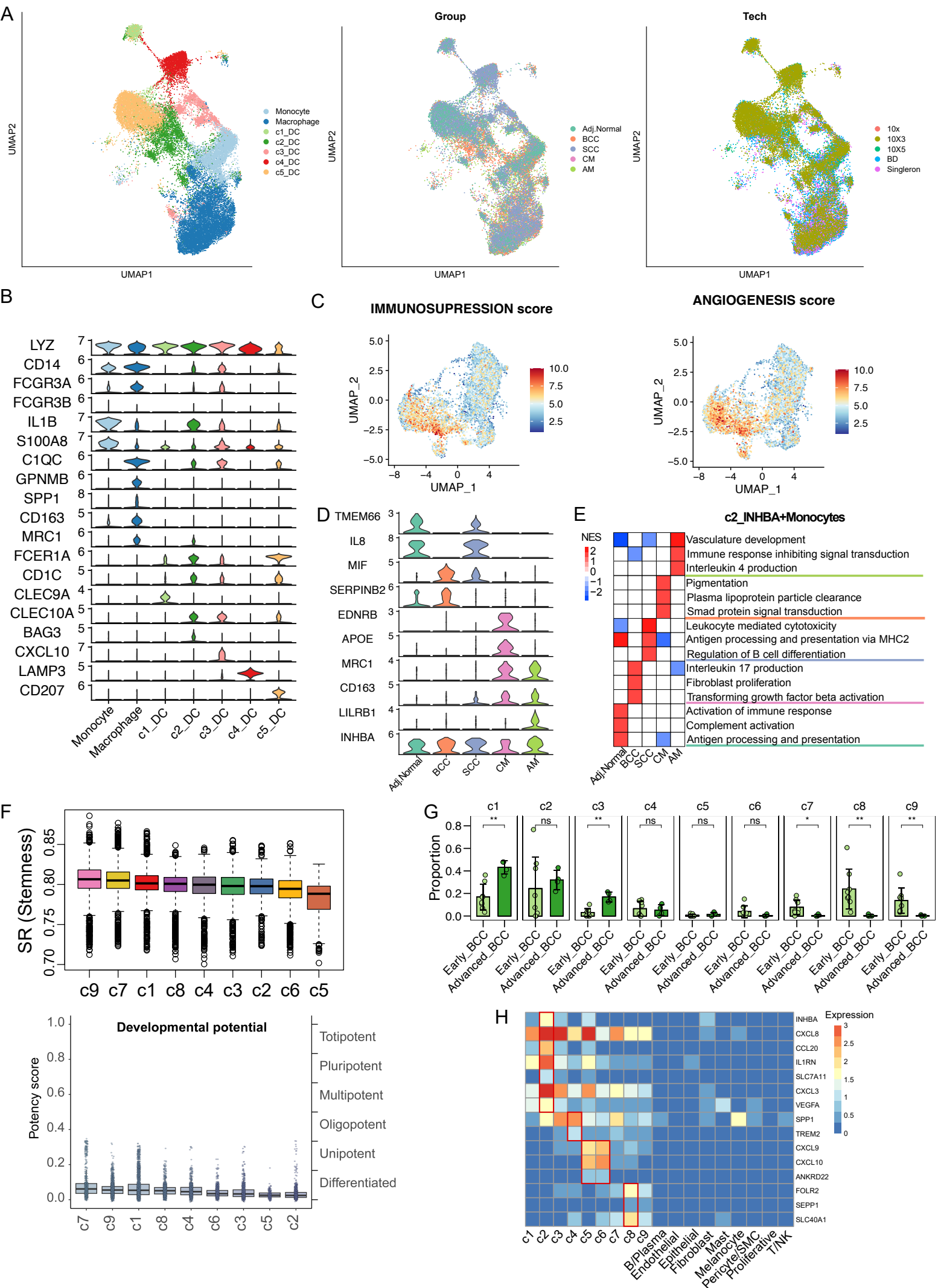

Supplement: Supplementary file 5 — Supporting Information [file CTM2-16-e70611-s005.pdf]

Figure S4

A

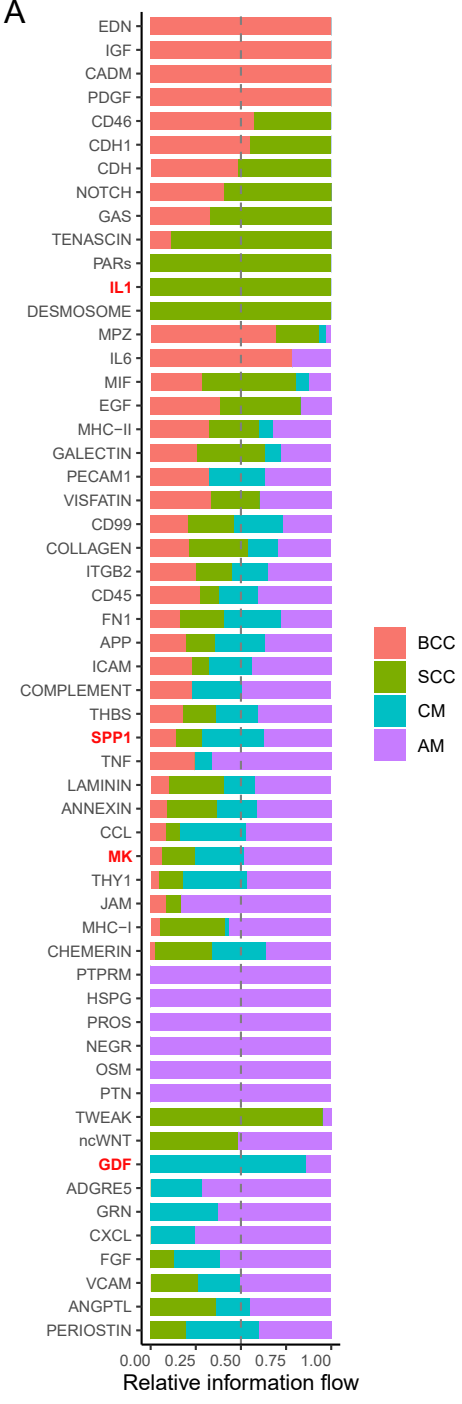

B

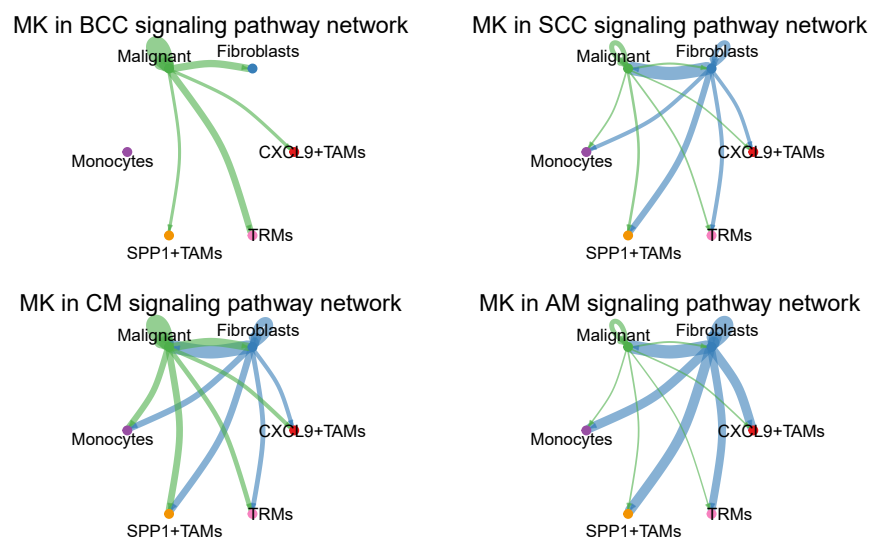

C

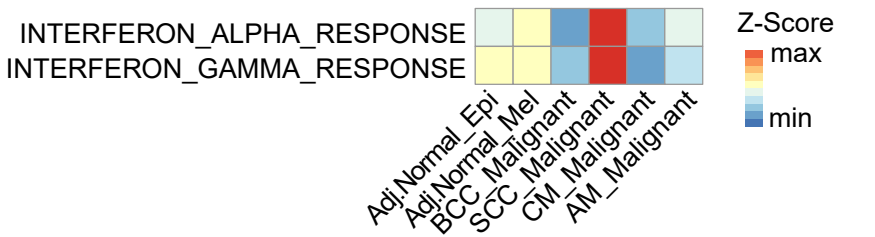

D

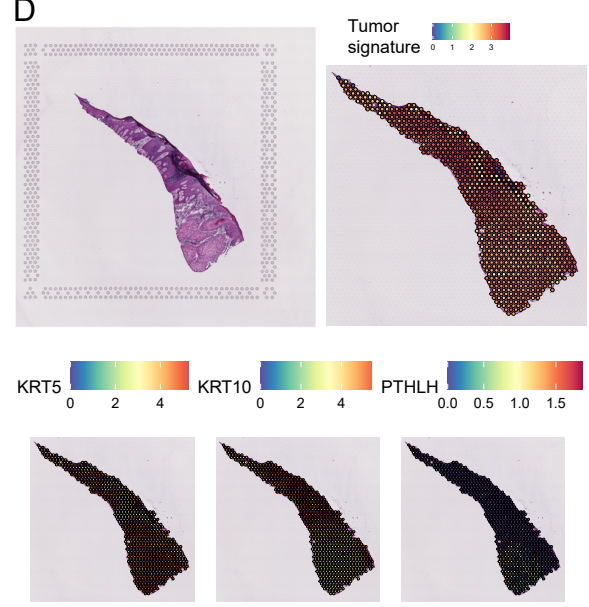

E

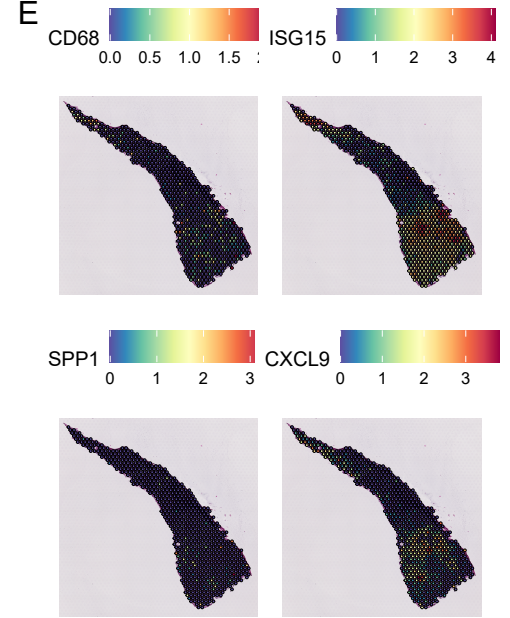

F

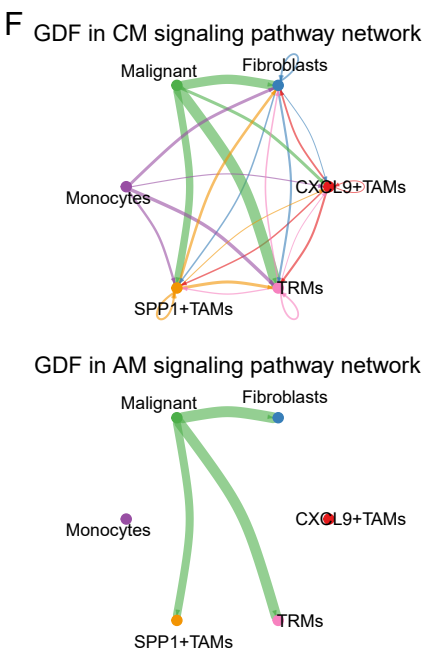

G

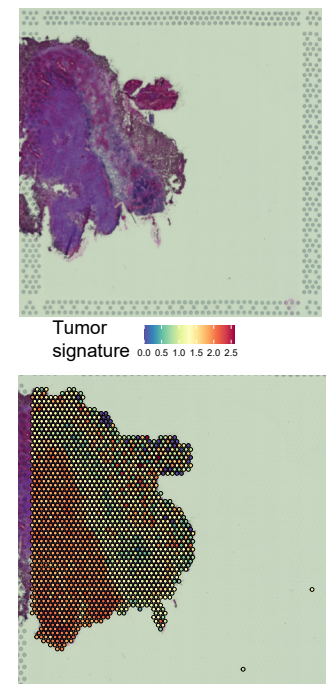

H

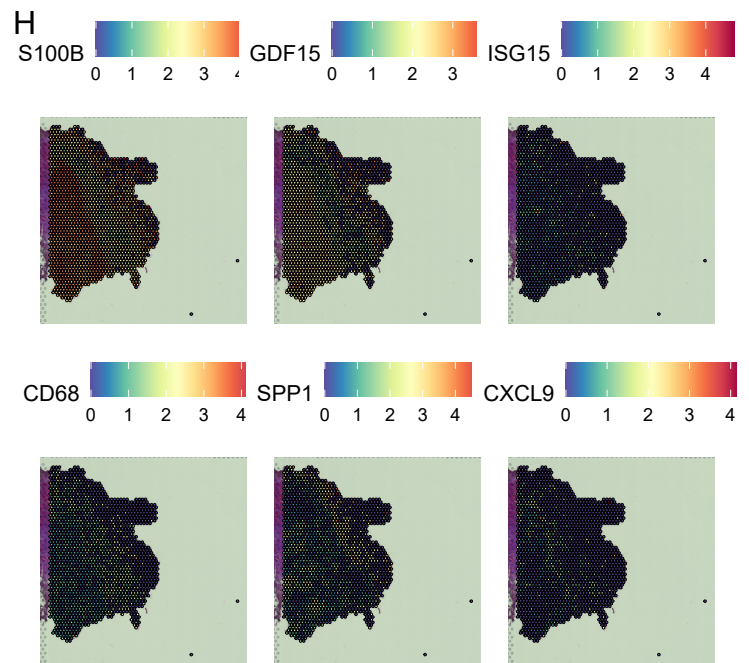

Supplement: Supplementary file 6 — Supporting Information [file CTM2-16-e70611-s006.pdf]

Figure S5

A

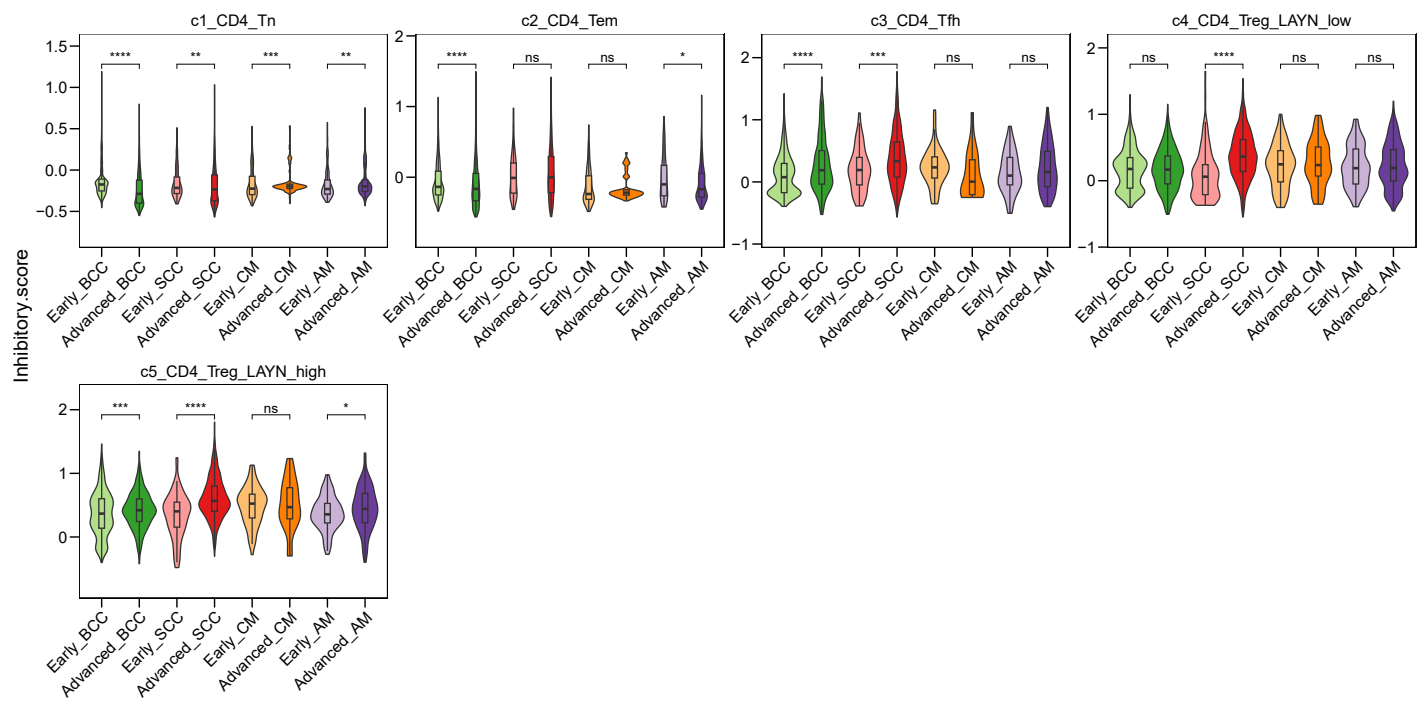

B

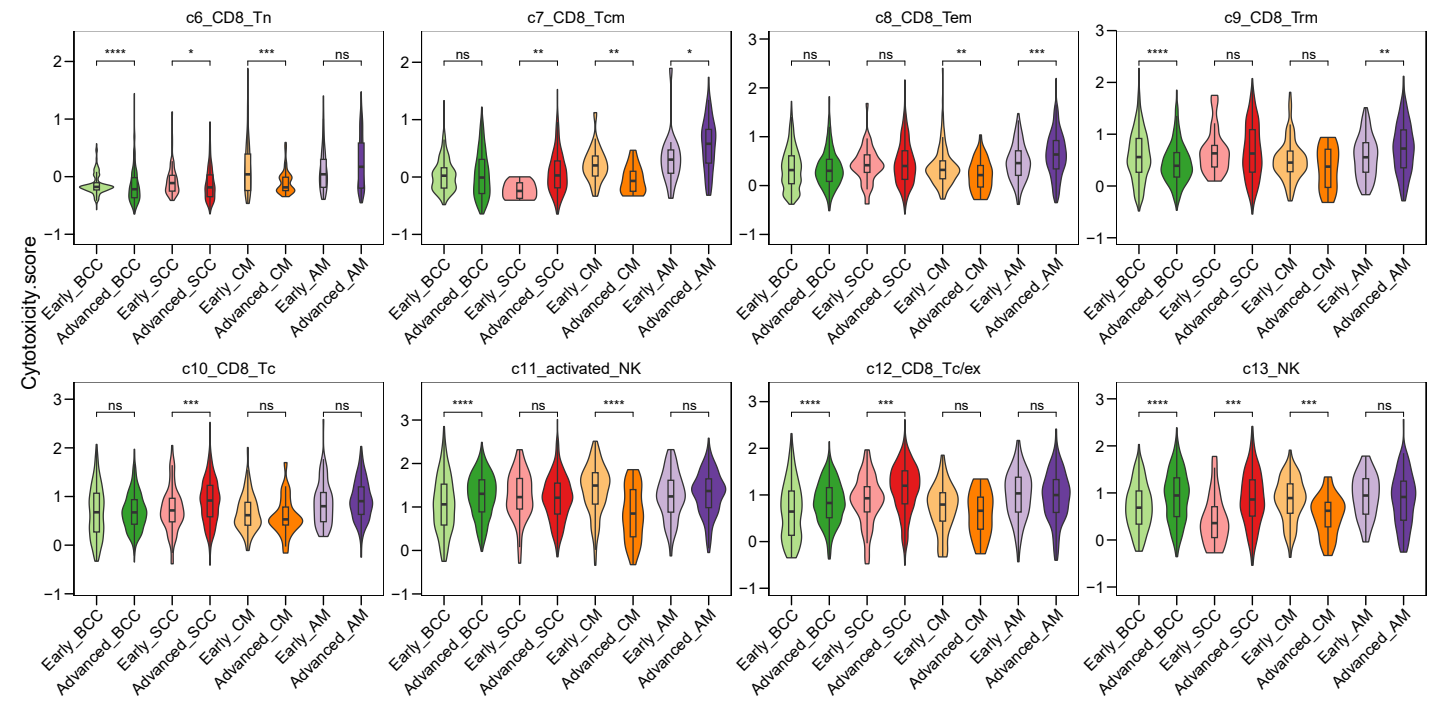

C

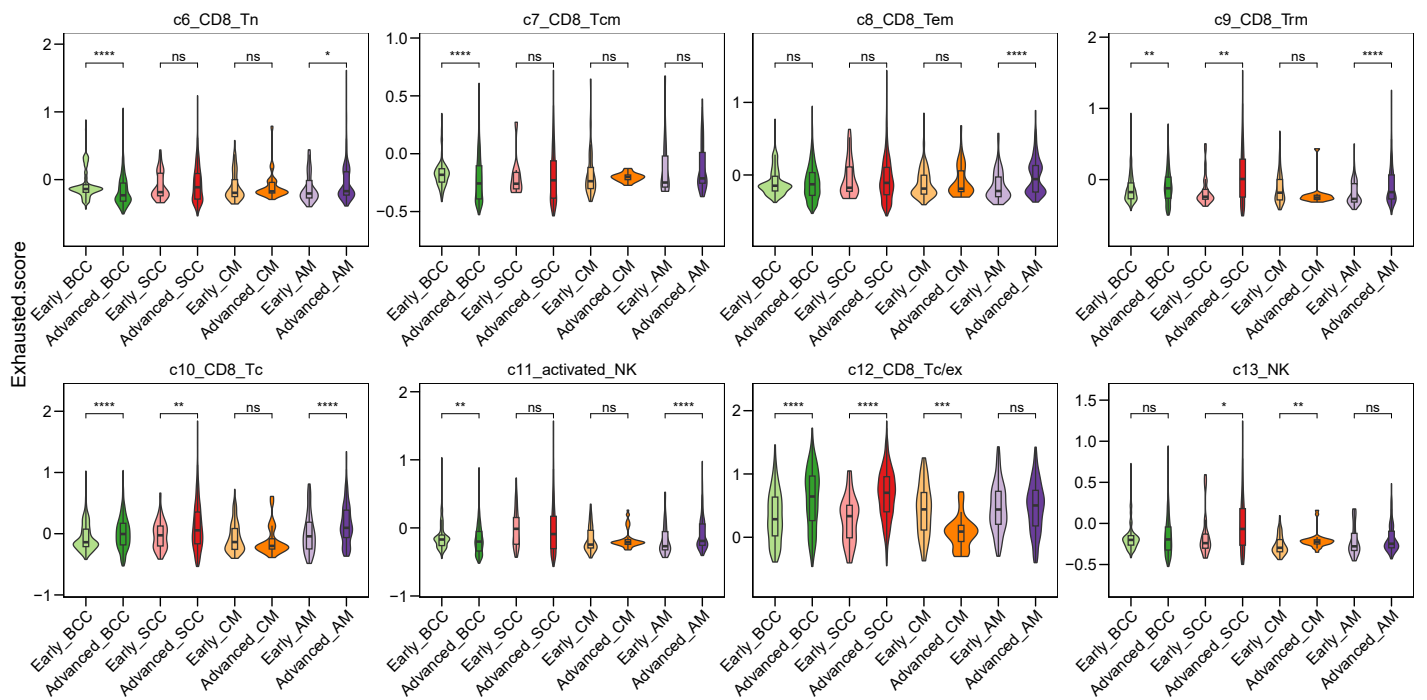

Supplement: Supplementary file 7 — Supporting Information [file CTM2-16-e70611-s009.pdf]

Figure S6

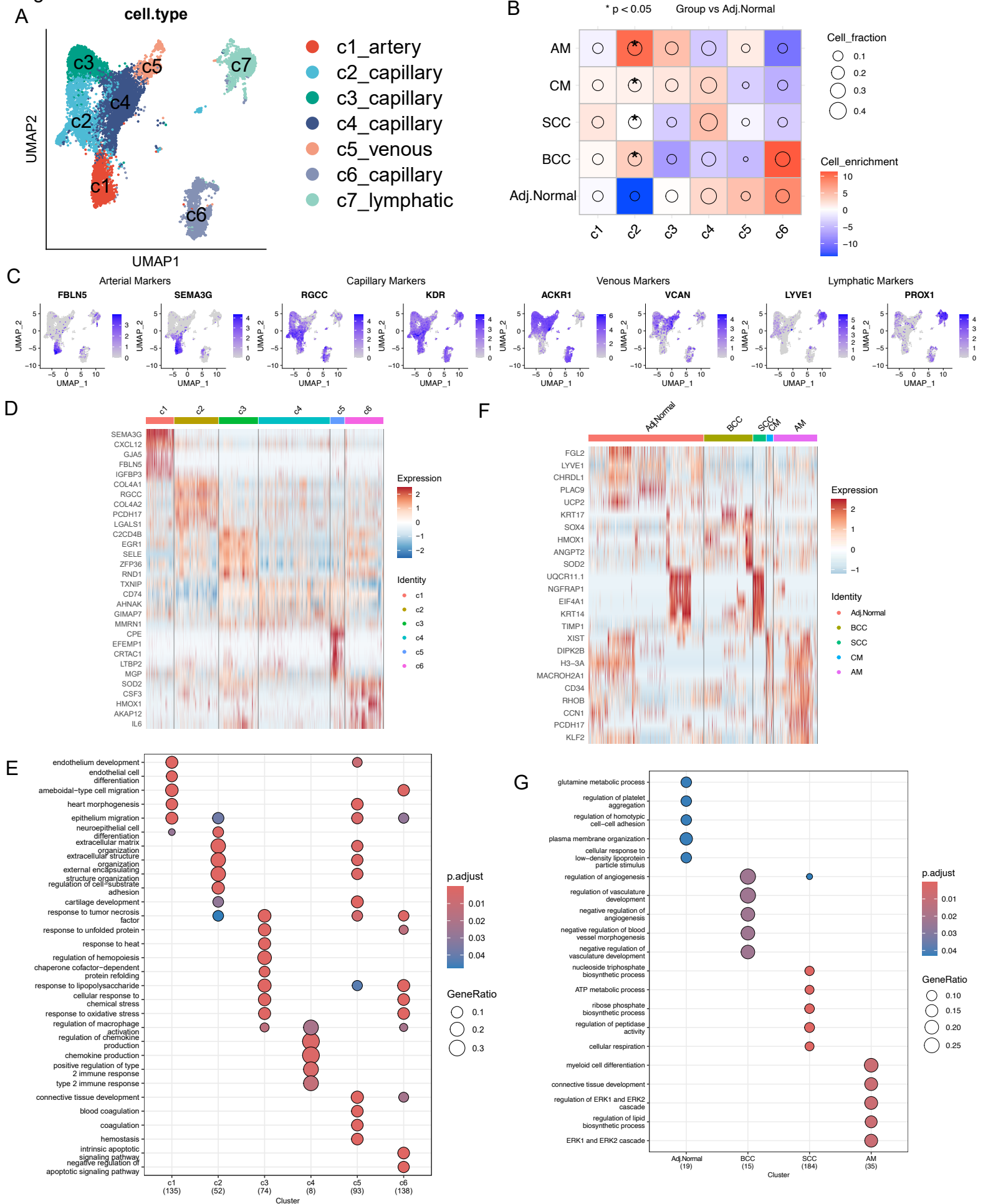

Supplement: Supplementary file 8 — Supporting Information [file CTM2-16-e70611-s004.pdf]

Figure S7

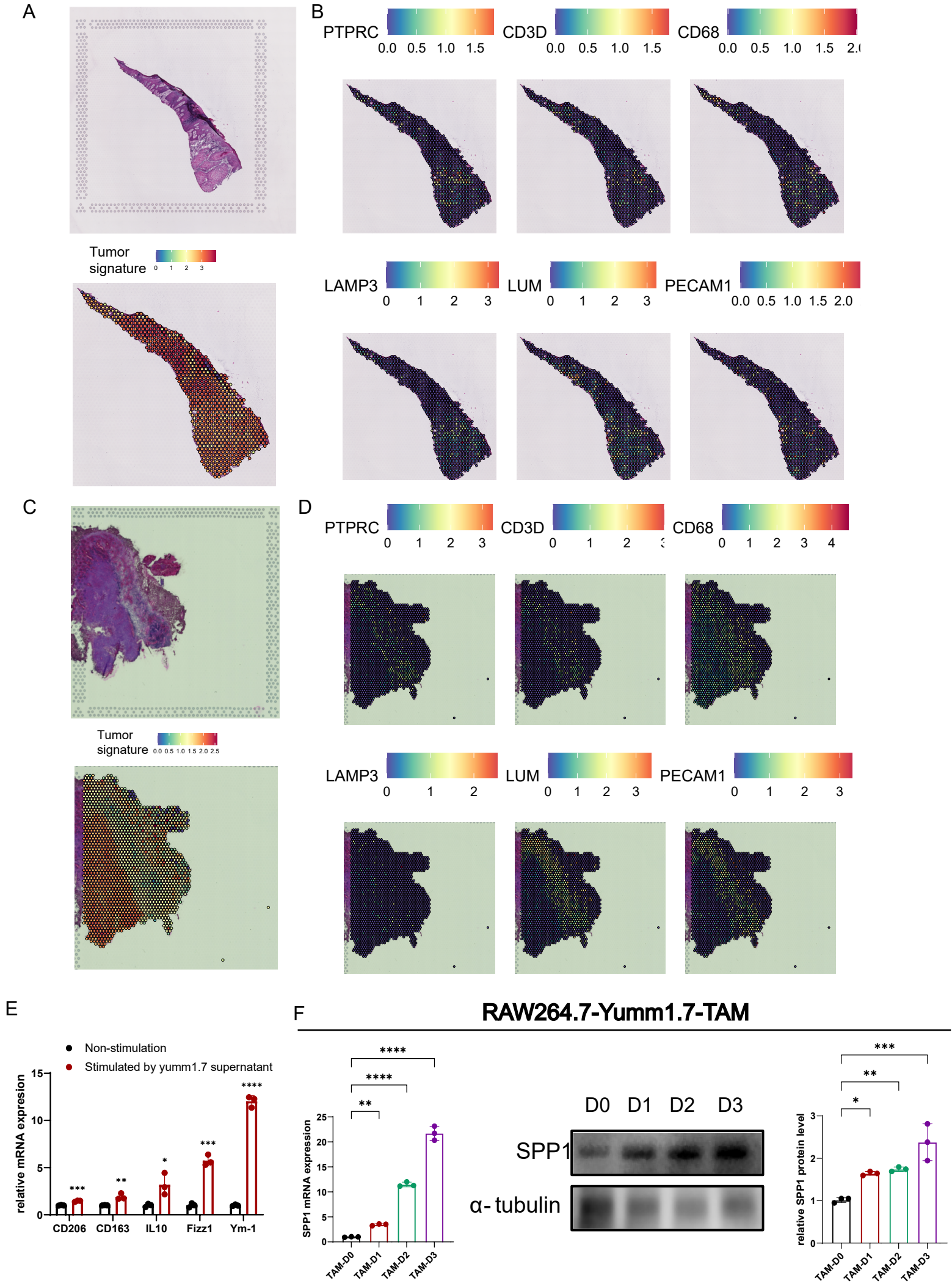

Supplement: Supplementary file 9 — Supporting Information [file CTM2-16-e70611-s007.pdf]
